# Computational quantum chemistry, molecular docking, and ADMET predictions of imidazole alkaloids of *Pilocarpus microphyllus* with schistosomicidal properties

**DOI:** 10.1371/journal.pone.0198476

**Published:** 2018-06-26

**Authors:** Jefferson A. Rocha, Nayra C. S. Rego, Bruna T. S. Carvalho, Francisco I. Silva, Jose A. Sousa, Ricardo M. Ramos, Ionara N. G. Passos, Josué de Moraes, Jose R. S. A. Leite, Francisco C. A. Lima

**Affiliations:** 1 The Postgraduate Program of the Northeast Network of Biotechnology, RENORBIO, Focal Point UFPI, Teresina, Piauí, Brazil; 2 Research Group in Natural Sciences and Biotechnology, Federal University of Maranhão, CIENATEC / UFMA, Grajaú, MA, Brazil; 3 Research Group in Computational Quantum Chemistry & Pharmaceutical Planning, State University of Piauí, GPQQ&PF / UESPI, Teresina, PI, Brazil; 4 Research Laboratory in Information Systems, Department of Information, Environment, Health and Food Production, Federal Institute of Piauí, LAPESI / IFPI, Teresina, PI, Brazil; 5 Research Center for Neglected Diseases, Guarulhos University, NPDN / UNG, Guarulhos, SP, Brazil; 6 Area Morphology, Faculty of Medicine, *Campus* Darcy Ribeiro, University of Brasília, UnB, Brasília, DF, Brazil; Hunan University, CHINA

## Abstract

Schistosomiasis affects million people and its control is widely dependent on a single drug, praziquantel. Computational chemistry has led to the development of new tools that predict molecular properties related to pharmacological potential. We conducted a theoretical study of the imizadole alkaloids of *Pilocarpus microphyllus* (Rutaceae) with schistosomicidal properties. The molecules of epiisopiloturine, epiisopilosine, isopilosine, pilosine, and macaubine were evaluated using theory models (B3lyp/SDD, B3lyp/6-31+G(d,p), B3lyp/6-311++G(d,p)). Absorption, distribution, metabolization, excretion, and toxicity (ADMET) predictions were used to determine the pharmacokinetic and pharmacodynamic properties of the alkaloids. After optimization, the molecules were submitted to molecular docking calculations with the purine nucleoside phosphorylase, thioredoxin glutathione reductase, methylthioadenosine phosphorylase, arginase, uridine phosphorylase, Cathepsin B1 and histone deacetylase 8 enzymes, which are possible targets of *Schistosoma mansoni*. The results showed that B3lyp/6-311++G(d,p) was the optimal model to describe the properties studied. Thermodynamic analysis showed that epiisopiloturine and epiisopilosine were the most stable isomers; however, the epiisopilosine ligand achieved a superior interaction with the enzymes studied in the molecular docking experiments, which corroborated the results of previous experimental studies on schistosomiasis.

## Introduction

Schistosomiasis is a neglected disease caused by the parasitic trematode *Schistosoma*. Reported in over 70 tropical and subtropical countries or territories, schistosomiasis is known to affect approximately 240 million people, and more than 700 people live in endemic areas [1]. Infection is prevalent in poorer communities throughout tropical and subtropical areas. Praziquantel, the only drug available to control the disease, was developed in the 1970s and has been shown to be ineffective against the larval stage of the parasite, with concerns about drug resistance. The search for new drugs, mainly from natural resources [2–3], is therefore of interest.

The interdisciplinary study of neglected diseases and medicinal chemistry is a new area of research that involves the rational planning, evaluation, and synthesis of new drugs in addition to the interpretation of their mode of action at the molecular level and the determination of biological effects arising from their molecular structures [4]. To complement this area of study, computational quantum chemistry has developed new tools to enable modeling and molecular dynamics studies that simulate biological tests and create new possibilities for drug models, without the need for solvent and reagent wastage [5–9].

Molecular docking is widely used to predict protein-ligand complexes and to screen large libraries for molecules that will modulate the activity of a biological receptor. Molecular docking remains an important tool for structure-based screening to find new ligands and chemical probes, it has enriched hit-rates and often confirming the predicted geometries of the docked complex [10–11]. The importance of optimizing the absorption, distribution, metabolism, excretion, and toxicity (ADMET) properties of compounds in addition to pharmacology increase drug discovery success. This sufficiently acceptable toxicity properties to success through the of human Phase I clinical trials. Intrinsic properties of the molecules and it is responsibility of the medicinal chemistry to optimize not only the pharmacological properties but also the drug-like properties of these molecules [12–13].

Among the candidates for new drugs are the imidazole alkaloids present in the species *Pilocarpus microphyllus* Stapf ex Holm. (Rutaceae), which have been noted in the literature for their pharmacological action against schistosomiasis [14–17]. However, little is known about their mode of action. These alkaloids, which are secondary metabolites found mainly in plants, mainly function as a defense against predators and pathogens.

Of the *P*. *microphyllus* alkaloids used in this study, epiisopiloturine (EPI) is the best characterized. EPI was identified in the 1970s [18] and its subsequent isolation and chemical characterization revealed strong pharmacological potency against *S*. *mansoni* [17,19,20–21]. Other biological functions have been described, including anti-inflammatory, anti-contraceptive [22], and gastro-protective effects [23]. The pharmacological action of epiisopilosine (EPIIS) and isopilosine (ISOP) against *S*. *mansoni* has also been described [16]. Macaubine (MAC) is the only alkaloid to show no effect against schistosomiasis [16], although pilosine (PILO) has not yet been tested.

Among these alkaloids in this study, little is known about their mechanism of action against *S*. *mansoni*; how these molecules interact with the possible enzymes of the worm, which pharmacological reactions are triggered; and how their chemical structures provide effective biological activity against this disease. As there is only a single drug used for treatment, with a high concern about associated resistance, we were motivated to apply the potential of computational quantum chemistry to the problem of this neglected disease. Thus, the main objective of the paper is to perform a theoretical study, in density functional theory (DFT) level, with geometry, electronic, and vibrational properties, molecular docking and absorption, distribution, metabolization, excretion, and toxicity (ADMET) predictions of the imizadole alkaloids of *P*. *microphyllus* with schistosomicidal properties.

## Materials and methods

### Computational details

Alkaloid geometry, electronic, and vibrational properties were studied using the program Gaussian 09 [24]. The GaussView 5.0.8 [25] software was used to obtain 3D structural models. Geometric optimization calculations were performed in accordance with DFT [26] by combining the B3LYP [27–28] functional hybrid and basis sets SDD, 6–31+g(d,p), and 6–311++g(d,p) [29–30]. Frequency calculations were performed to obtain thermodynamic properties and to verify that each optimization achieved an energy minimum.

The quantum chemical descriptors extracted directly from the Gaussian output file were Mulliken, NBO and ChelpG charge, electronic density, dipole moment, the energy of the highest occupied molecular orbital (E_HOMO_), and the energy of the lowest unoccupied molecular orbital (E_LUMO_) [31–32]. All calculations were performed in the gas phase. Time-dependent density functional theory (TDDFT) was used to calculate the energies and intensities of electronic transitions [33–34]. The calculated electronic transitions, infrared, and Raman spectra were convoluted by using Gaussian functions with half-widths of 25.000 cm^-1^ computed by the Swizard program [35].

### ADMET predictions

The prediction of pharmaceutical parameters was conducted using the freely available pre-ADMET® and FAF-Drugs4® software packages. The in sílico methodology used with the EPI, EPIIS, ISOP, PILO, and MAC molecules included physical–chemical parameters, drug-likeness profile, pharmacokinetic profile (ADME), and toxicity. Among the most relevant parameters of absorption were the observation of the ability of the drug to cross the blood-brain barrier (BBB), as well as the drug absorption rate (Caco2), the rate of absorption by human intestinal cells (HIA) and excretion (MDCK). Regarding the metabolization process, the capacity of inhibition, non-inhibition and substrate formation by the molecules through their behavior on CYP-450 subfamilies was evaluated [12–13].

### Molecular docking

The 3D structures of all possible *S*. *mansoni* targets were obtained from the Protein Data Bank (PDB) [36] with the codes (3QSD, 4Q3P, 4CQF, 4L5A, 1TCU, 2V6O, and 4TXH). All docking procedures utilized the Autodock 4.2 package [37–39]. Protein (ACE) and ligands were prepared for docking simulations with AutoDock Tools (ADT) version 1.5.6. [40]. The receptor was considered rigid; each ligand was considered flexible. Gasteiger [41] partial charges were calculated after the addition of all hydrogens. Nonpolar hydrogen atoms of the protein and ligand were subsequently merged. A cubic box of 60 × 60 × 60 points with a spacing of 0.35 Å between the grid points was generated for the whole protein target. The affinity grid centers were defined on residue Asp158 for ARG, Ala118 for PNP, Asp230 for MTAP, Tyr341 for HDAC8, Gln201 for UP, Tyr296 for TGR, and Cys100 for 2CB1. The global search Lamarckian genetic algorithm (LGA) [42] and the local search (LS) pseudo-Solis and Wets [43] methods were applied in the docking search. Each ligand was subjected to 100 independent runs of docking simulations [44]. Other docking parameters were set as the default values. The resulting docked conformations were clustered into families according to the RMSD. For a more detailed analysis, the coordinates of the selected complexes were chosen by the criterion of lowest docking conformation of the cluster with lowest energy in combination with a visual inspection.

## Results and discussion

### Computational details

**Fig 1:**
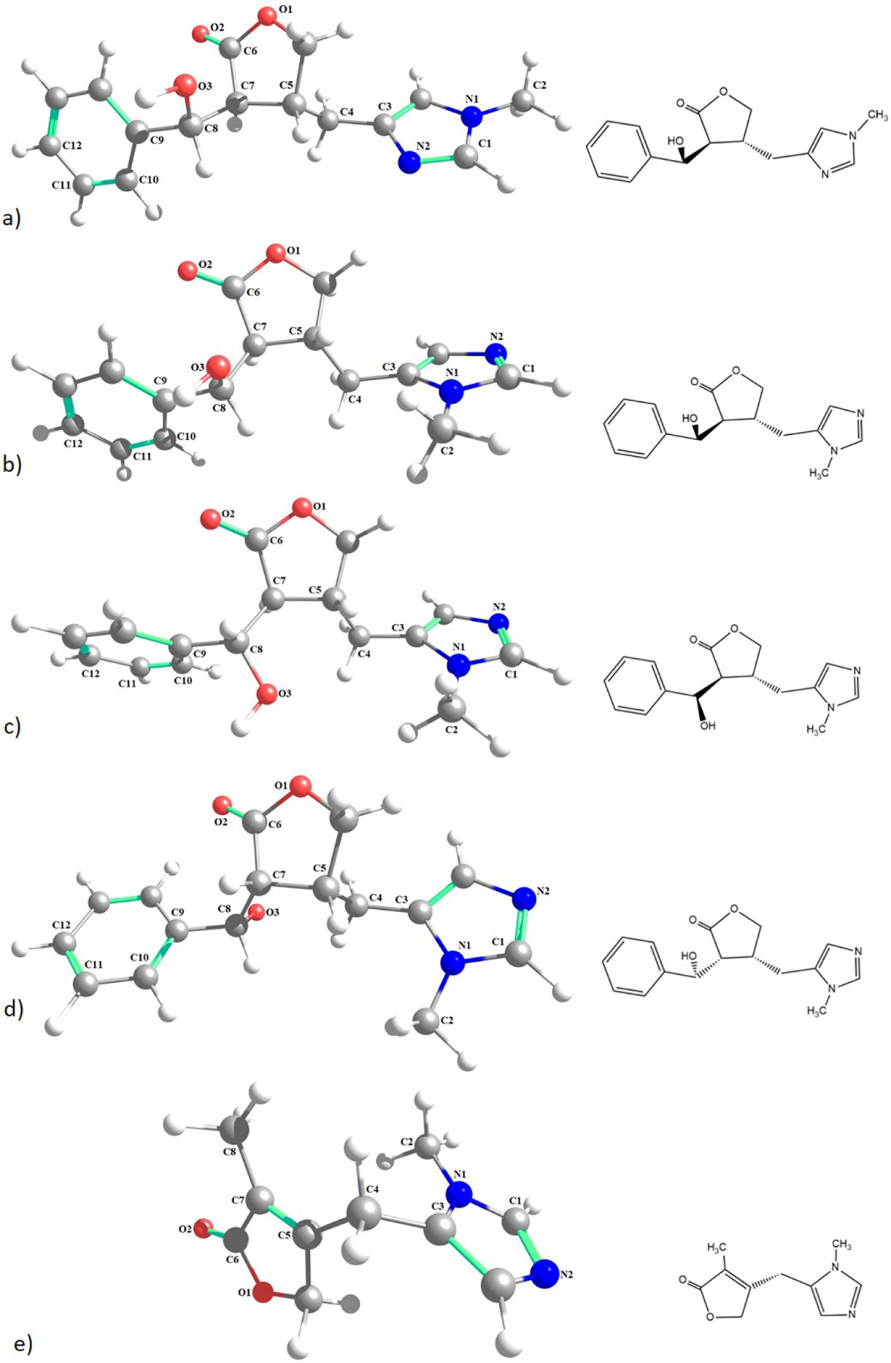
Optimized chemical structures of alkaloids. a) epiisopiloturine, b) epiisopilosine, c) isopilosine, d) pilosine, and e) macaubine.

The imidazole alkaloids used in this study have the chemical structure shown in Fig 1. It is possible to observe four isomeric forms between the EPI, EPIIS, ISOP, and PILO alkaloids (C_16_H_18_N_2_O_3_), and the presence of optical isomerism between the EPIIS, ISOP, and PILO alkaloids, which is expressly observed in the rotation of the C4 and C9 atoms (conformers), and in the chiral carbons C5, C7, and C8, which corroborate with data previously presented in experimental studies [16].

#### Charges study

The atomic charges of the alkaloids under study are shown in Table 1 for the theoretical model B3lyp/6-311++G(d,p). The bond between C2-N1 is covalent, so electrons are shared between them. However, the nitrogen atom is more electronegative and attracts more electronic density to itself, which concentrates the electron density. The NBO loads in all binders (EPI, EPIIS, ISOP, PILO, and MAC) had negative charge values, whereas for the Chelpg and Mulliken methods, the loads were predicted to be positive (Table 1). All other models are shown in S1 and S2 Tables.

**Table 1:** Atomic charges (in atomic unit, a.u.) by the Mulliken, Chelpg, and NBO methods of the epiisopiloturine, epiisopilosine, isopilosine, pilosine and macaubine alkaloids using the theoretical model B3lyp/6-311++G(d,p).

	EPI			EPIIS			ISOP			PILO			MAC		
	Chelp	NBO	Mull	Chelp	NBO	Mull	Chelp	NBO	Mull	Chelp	NBO	Mull	Chelp	NBO	Mull
**N1**	0.105	-0.406	0.017	0.063	-0.415	0.005	0.115	-0.415	0.014	0.113	-0.417	0.005	0.098	-0.414	-0.003
**N2**	-0.577	-0.513	0.096	-0.562	-0.492	-0.104	-0.572	-0.493	-0.099	-0.592	-0.492	-0.110	-0.543	-0.490	-0.107
**O1**	-0.456	-0.547	-0.009	-0.429	-0.544	-0.016	-0.414	-0.543	-0.023	-0.441	-0.547	-0.025	-0.452	-0.543	-0.088
**O2**	-0.572	-0.576	-0.256	-0.526	-0.564	-0.291	-0.522	-0.565	-0.284	-0.545	-0.566	-0.275	-0.548	-0.570	-0.307
**O3**	-0.564	-0.746	-0.207	-0.593	-0.741	-0.210	-0.541	-0.751	-0.142	-0.568	-0.735	-0.177	-	-	-
**C1**	0.115	0.218	0.259	0.188	0.211	0.081	0.197	0.208	0.113	0.190	0.208	0.077	0.174	0.214	0.085
**C2**	-0.163	-0.347	-0.316	-0.276	-0.352	-0.303	-0.371	-0.352	-0.325	-0.370	-0.350	-0.293	-0.277	-0.353	-0.389
**C3**	0.439	0.108	0.175	-0.062	0.109	0.467	-0.153	0.112	0.592	-0.182	0.114	0.178	-0.130	0.109	0.481
**C4**	-0.377	-0.401	-0.984	-0.234	-0.410	-0.694	-0.056	-0.417	-0.792	-0.010	-0.428	-0.341	0.068	-0.447	-1.043
**C5**	0.223	-0.242	0.079	0.237	-0.240	0.005	0.187	-0.231	-0.095	0.099	-0.223	0.164	0.015	0.024	0.607
**C6**	0.695	0.817	-0.221	0.639	0.828	-0.060	0.614	0.818	-0.206	0.667	0.822	-0.002	0.741	0.775	-0.071
**C7**	0.114	-0.319	-0.182	0.123	-0.318	0.082	0.067	-0.318	0.285	0.221	-0.325	-0.463	-0.169	-0.129	0.335
**C8**	0.145	0.139	0.298	0.108	0.132	0.152	-0.073	0.133	-0.284	-0.014	0.130	0.183	-0.102	-0.600	-0.545
**C9**	0.091	-0.075	0.767	0.104	-0.075	0.515	0.192	-0.075	1.016	0.156	-0.077	0.706	-	-	-
**C10**	-0.177	-0.199	-0.319	-0.163	-0.202	-0.034	-0.158	-0.201	-0.231	-0.161	-0.202	-0.110	-	-	-
**C11**	-0.051	-0.200	-0.311	-0.058	-0.200	-0.432	-0.076	-0.198	-0.511	-0.083	-0.200	-0.451	-	-	-
**C12**	-0.118	-0.200	-0.446	-0.114	-0.199	-0.339	-0.106	-0.196	-0.382	-0.100	-0.199	-0.351	-	-	-

The observation of the bond between O2 = C6-O1 revealed that the charges were not well described using the Mulliken method as there was consistency between these charges, and because they have negative values for all atoms in the bond, including C7. The nature of the type of binding may be the determining factor in these charges, which the Mulliken method cannot accurately describe (Table 1). The NBO method again described the load on this connection.

The bond length C8-O3 showed negative values for all ligands for the O3 atom in the three types of charges tested. In contrast, C8 always presented NBO with a positive charge, independent of the isomer. However, in the ISOP and PILO isomers with the Chelpg and Mulliken methods, negative charges were presented which did not corroborate with the NBO and the electronegativity of the atoms themselves (Table 1).

#### Atomic distances analysis

The binding lengths and binding angles calculated in the three theoretical models showed a small decrease in values with an increase in the number of base functions (S3 Table), except for the EPI alkaloid, which had the same values in B3lyp/6-31+G(d,p) and B3lyp/6-311++G(d,p) (Table 2).

**Table 2:** Distances of the atomic bonds (Å), atomic angles, and dihedral angles (°) of the alkaloids epiisopiloturine, epiisopilosine, isopilosine, pilosine and macaubine using the theoretical model B3lyp/6-311++G(d,p).

B3lyp/6-311++G(d,p)	EPI	EPIIS	ISOP	PILO	MAC
N1 –C2	1.454	1.454	1.455	1.453	1.455
N2 –C1	1.317	1.311	1.312	1.311	1.312
O2 = C6	1.207	1.196	1.198	1.197	1.201
O1 –C6	1.356	1.360	1.356	1.360	1.371
O3 –C8	1.432	1.429	1.440	1.427	-
N1 –C1 –N2	112.3	112.3	112.3	112.3	112.3
C1 –N1 –C2	126.9	125.7	125.8	125.7	126.0
C3 –C4 –C5	113.3	114.5	114.3	113.7	114.3
O2 = C6—O1	122.2	121.9	122.1	121.9	122.7
O3 –C8 –C7	106.3	106.5	106.3	108.0	-
C7 –C8 –C9	115.1	115.4	114.1	114.3	-
C8 –C9 –C10	119.2	119.0	120.4	119.1	-
C4 –C5 –C7 –C8	140.7	82.4	88.2	41.2	-
C5 –C7 –C8 –O3	63.3	62.2	−45.0	−75.1	-

Among the alkaloids studied, a highly regular chemical structure was observed in the imidazolic ring, with similar distances for N1-C2 and N2-C1 and similar angles for N1-C1-N2 and C1-N1-C2; only EPI had a slight variation of 1°.

In the dihydrofuran ring, the atomic angles between O = C6-O1 were similar in EPI, ISOP, and MAC (122.2°), and between EPIIS and PILO (121.9°). The distance of the atomic double bond between O2 = C6 was higher in EPI (1.207 Å) and lower in EPIIS (1.196 Å). The length of the O1-C6 bond had similar values in EPIIS and PILO (1.360 Å) and EPI and ISOP (1.356 Å). In the benzene ring, the internal angles were highly similar, with only a small variation of 1° in ISOP.

Through the observation of the chiral C8, which links the benzene ring to the dihydrofuran ring, it was observed that the O3-C8 distance was higher in ISOP (1.440 Å) and lower in PILO (1.427 Å). The angle between O3-C8-C7 was also higher in PILO (108.0°), but similar in the other alkaloids (106.3°) and (106.5°). In addition, it was possible to observe an expressive variation in the dihedral angle in the C4-C5-C7-C8 atoms between the optically symmetric EPIIS (82.4°), ISOP (88.2°), and PILO (41.2°) and the C5-C7-C8-O3 atoms of EPIIS (62.2°), ISOP (−45.0°), and PILO (−75.1°) (Table 2).

The experimental X-ray results of a macrophage of the MAC alkaloid were defined [16], where similarities in the distances of the chemical bonds presented in our theoretical calculations were found in N1-C2 (1.448 Å), N2-C1 (1.318 Å), O2 = C6 (1.210 Å), C6-O1 (1.350 Å), N1-C1-N2 (108.3°).

#### Relative energies study

**Fig 2:**
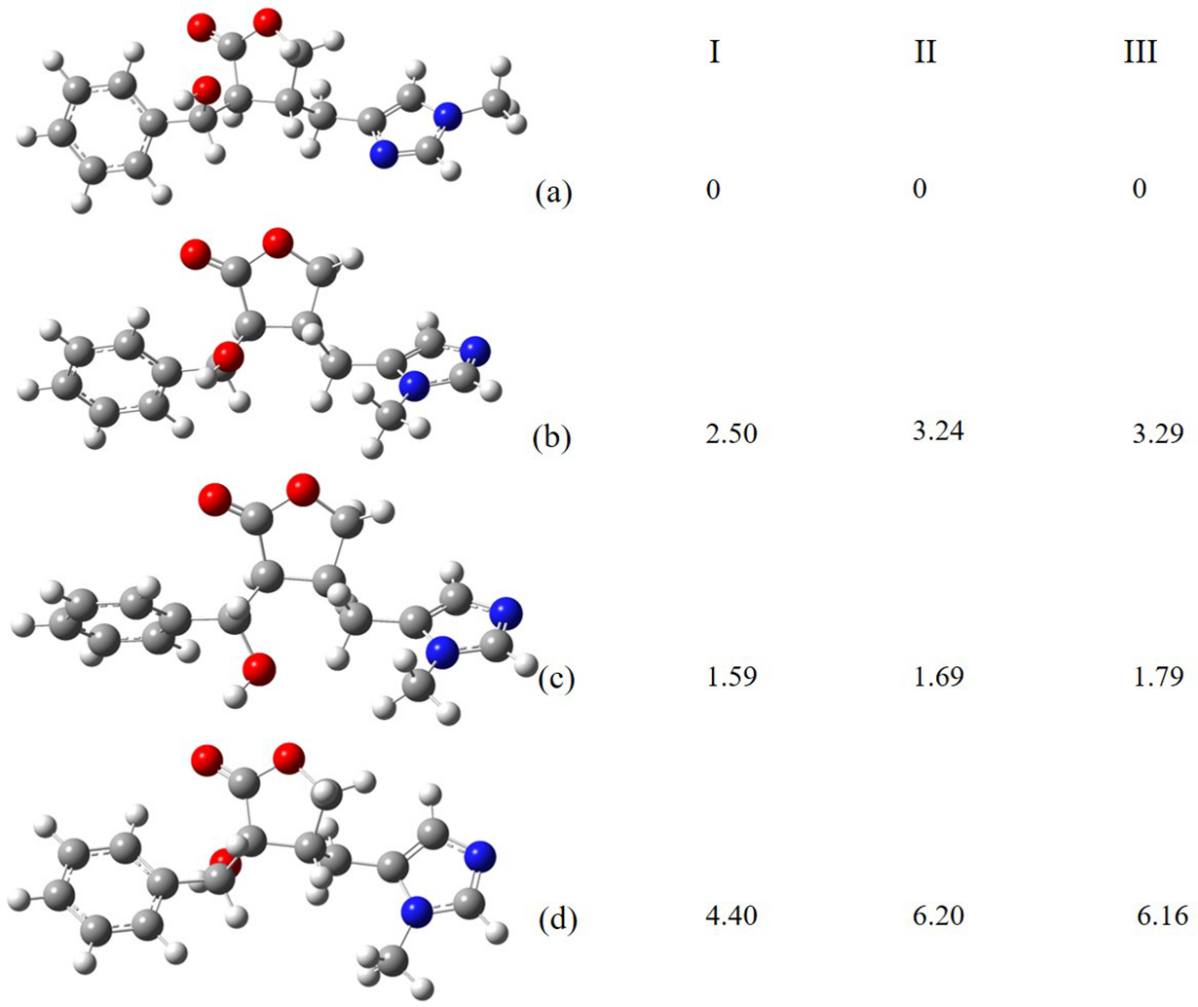
Relative energies in (kcal mol^-1^) of alkaloids isomers of EPI by theoretical models B3lyp/SDD (I), B3lyp/6-31+G(d,p) (II), and B3lyp/6-311++G(d,p) (III). a) epiisopiloturine, b) epiisopilosine, c) isopilosine and d) pilosine.

The relative energies between the isomeric alkaloids demonstrated that the EPI alkaloid was more stable than the others compounds (Fig 2; S4 Table). In the three theoretical models, the energy ranking was similar. However, as expected, the B3lyp/6-311++G(d,p) model showed that EPI was more stable between isomers compared with ISOP, by approximately 1.79 kcal mol^-1^, followed by EPIIS (3.29 kcal mol^-1^) and PILO (6.16 kcal mol^-1^). The stability may suggest a greater number of alkaloids among the studied isomers. The chemical structure of these alkaloid isomers is evidence of the difference in their stability across the dihedral angles (Table 2); by acquiring a cis conformation, the PILO alkaloid tends to be less stable compared with EPI, which is the most stable, given of its trans configuration. In this way, chemical reactions induced by light, temperature, or electricity [45] could easily result in the conversion of these less-stable models to more trans models, such as ISOP, which is isomeric with PILO, by changing conformation.

#### Orbitals molecular predictions

**Fig 3:**
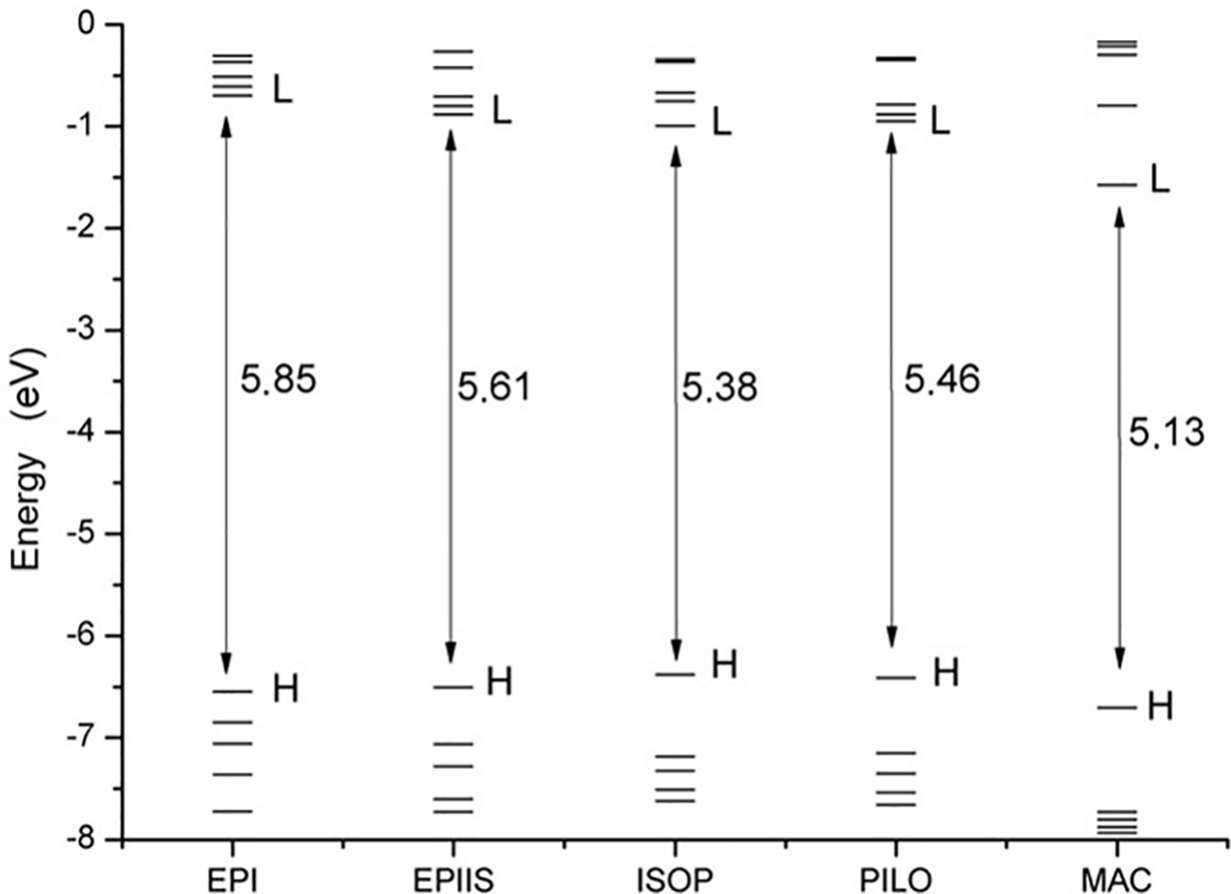
Graphic of the molecular orbitals HOMO and LUMO of the epiisopiloturine, epiisopilosine, isopilosine, pilosine and macaubine alkaloids using the theoretical model B3lyp/6-311++G(d,p).

The analysis of molecular orbitals showed a pattern in the values between alkaloids in the three models studied, with a small decrease in values in relation to the increase in the base functions (S5 Table). The analysis of the results obtained in the B3lyp/6-311++G(d,p) model revealed that the energy gaps were very close, with higher values of gap energy in EPI (5.85 eV) and smaller values in MAC (5.13 eV). The EPIIS, ISOP, and PILO optical isomers had gap values of 5.61 eV, 5.38 eV, and 5.46 eV, respectively (Fig 3).

The distance between LUMO+1 and HOMO-1 tended to increase in relation to the decrease in gap energy, from EPI to MAC. The proximity between the orbitals facilitates the interaction between the electrons, as observed in EPI (6.24 eV), EPIIS (6.26 eV), PILO (6.26 eV), ISOP (6.43 eV), and MAC (6.93 eV) (Fig 3). The molecular boundary orbitals HOMO and LUMO are responsible for the biological interactions between ligands and proteins [46–48]

**Fig 4:**
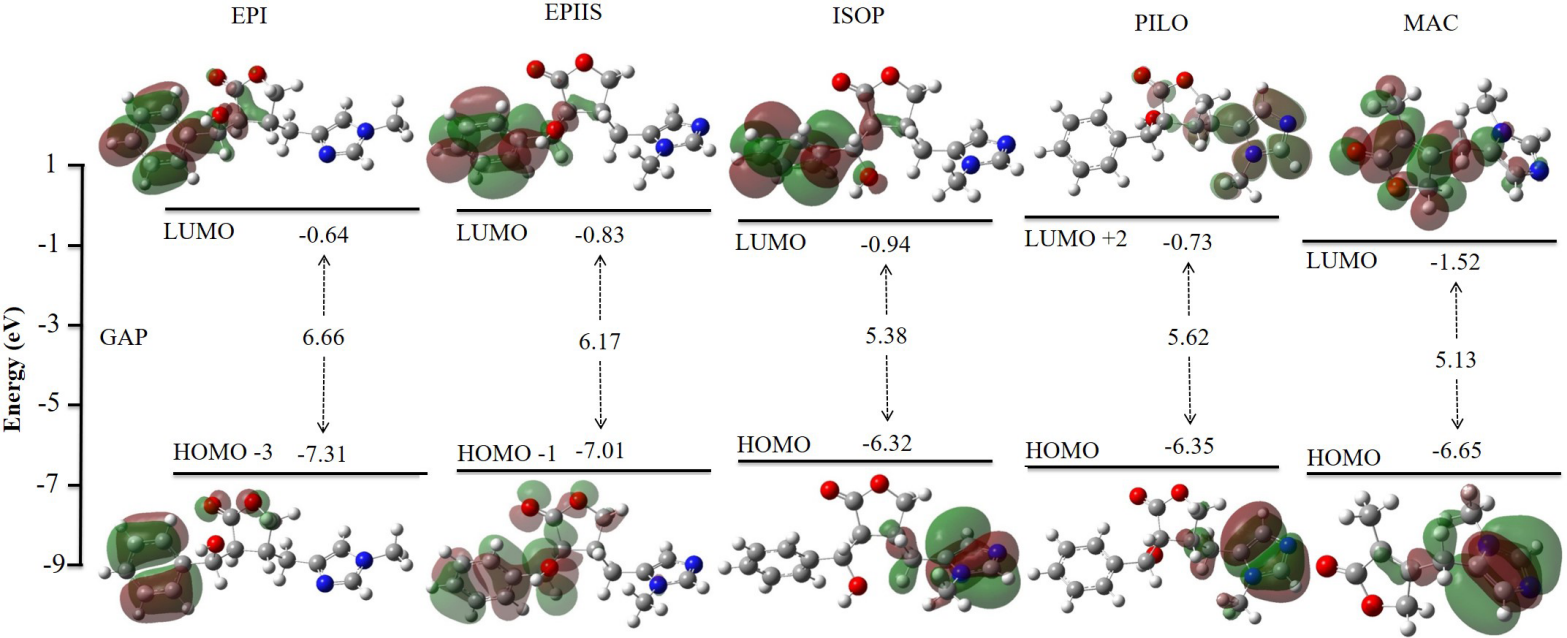
The molecular orbitals HOMO and LUMO of the epiisopiloturine, epiisopilosine, isopilosine, pilosine and macaubine alkaloids calculated using the theoretical model B3lyp/6-311++G(d,p) in the Swizard program.

The energy quantum jumps between the molecular orbits of the alkaloids were quantified (Table 3, Fig 4). For the EPI alkaloid, the highest value of the oscillator force was 0.0229 at a wavelength in the range of 230 nm and showed a jump between 73 (HOMO-3) and 77 (LUMO), with a gap of 6.66 eV. For the EPIIS alkaloid, the highest value of the oscillator force was 0.0150 at 237 nm and showed a jump between 75 (HOMO-1) → 77 (LUMO), with a gap of 6.17 eV. For the ISOP alkaloid, the highest value of the oscillator force was 0.0203 at 249 nm and showed a jump between 76 (HOMO) → 77 (LUMO), with a gap of 5.38 eV. For the PILO alkaloid, the highest value of the oscillator strength was 0.0034 at 236 nm and showed a jump between 76 (HOMO) → 79 (LUMO+2), with a gap of 5.62 eV. For MAC, the highest value of the oscillator force was 0.0139 and showed a jump between 51 (HOMO) → 52 (LUMO) with a gap of 5.13 eV (Table 3, Fig 4). This TD-DFT approach is considered reliable for describing geometries and spectral properties [49].

**Table 3:** Main assignments of the bands of the electronic spectrum of UV-Vis, energy, and types, calculated using the Swizard program for the molecular forms epiisopiloturine, epiisopilosine, isopilosine, pilosine and macaubine. I = imidazole, D = dihydrofuran, B = benzene.

Wavelength (nm) / Strength of the oscillator [f]	Composition	Energy (eV)	Type of charge transfer
EPI			
238 [0.0084]	75→79 (28%)	5.20	CT BD-I
237 [0.0021]	76→78 (95%)	5.23	CT I-B
230 [0.0229]	73→77 (18%)	5.38	CT BD-BD
EPIIS			
244 [0.0057]	76→78 (91%)	5.08	CT I-B
238 [0.0008]	76→77 (57%)	5.20	CT I-B
237 [0.0150]	75→77 (37%)	5.23	CT BD-B
ISOP			
249 [0.0203]	76→77 (91%)	4.96	CT I-B
243 [0.0002]	76→79 (62%)	5.10	CT I-I
237 [0.0042]	74→77 (37%)	5.23	CT I-B
PILO			
253 [0.0026]	76→77 (49%)	4.89	CT I-B
243 [0.0034]	76→78 (49%)	5.09	CT I-B
236 [0.0034]	76→79 (24%)	5.23	CT I-I
MAC			
281 [0.0139]	51→52 (98%)	4.40	CT I-D
253 [0.0004]	49→52 (38%)	4.90	CT I-D
237 [0.0010]	51→53 (97%)	5.23	CT I-I

**Fig 5:**
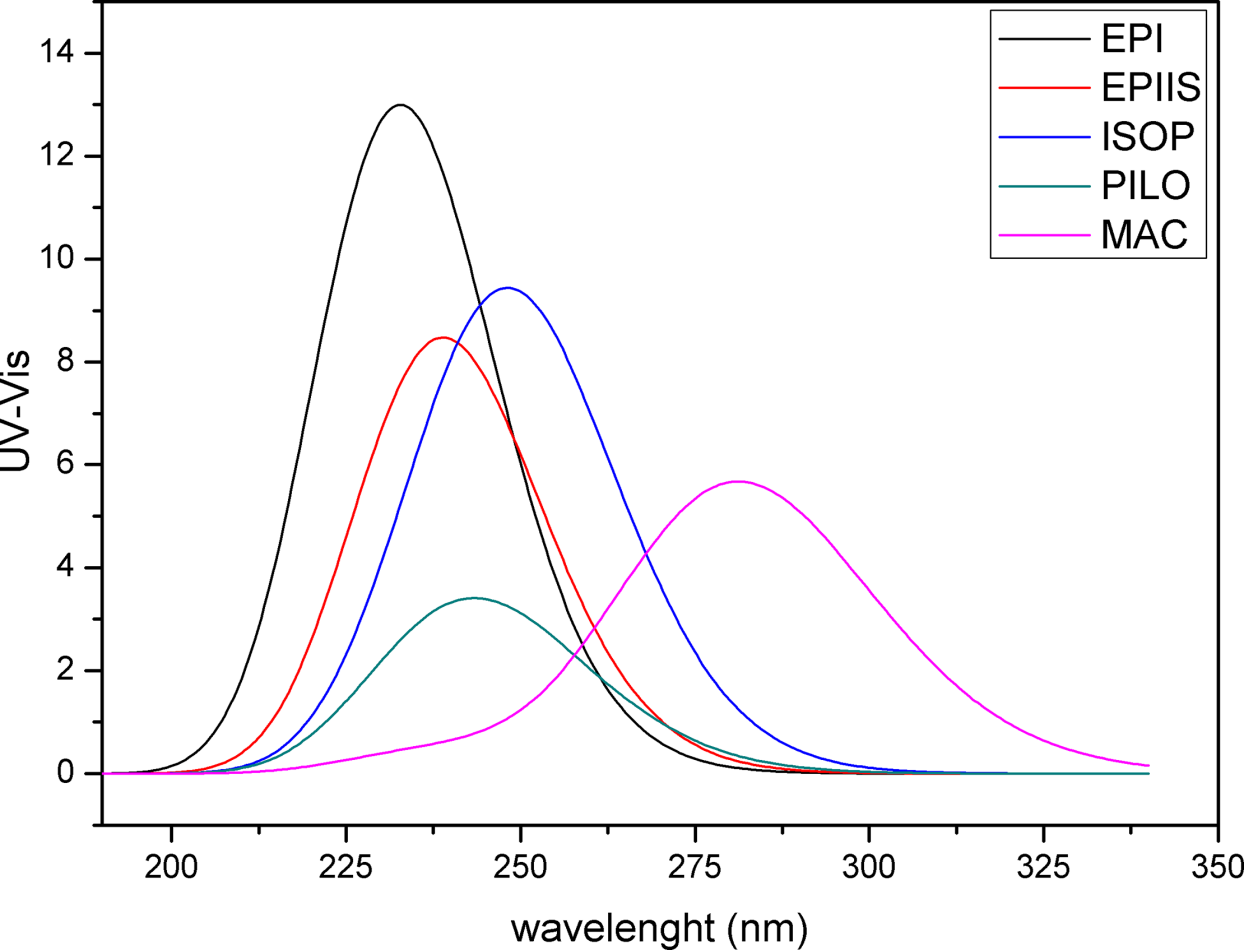
UV-Vis spectrum of the epiisopiloturine, epiisopilosine, isopilosine, pilosine and macaubine alkaloids using the theoretical model B3lyp/6-311++G(d,p).

The results obtained for the energy jumps corroborated with the results obtained from the UV-Vis spectra (Fig 5), where the graph peak represents the same values of the molecular orbitals. Spectroscopic UV-Vis data showed absorption bands at 230 nm for EPI, 237 nm (EPIIS), 249 nm (EPIIS), 243 nm (PILO), and 281 nm (MAC) using the theoretical model B3lyp/6-311++G(d,p). The theoretical model B3lyp/6-31+G(d,p) was highly similar, and the B3lyp/SDD model showed a more distant wavelength than these two models (S1 Fig).

#### Spectral analysis

The infrared spectra are shown in S8 Table. Similarities can be observed in the vibrational frequencies between the EPI, EPIIS, ISOP, and PILO alkaloids, which demonstrate the isomeric conformations (Figs (a) and (b) in S2 Fig. The symmetric carbonyl stretching (C = O) showed a strong peak in the regions of 1785 cm^-1^, 1846 cm^-1^, 1838 cm^-1^, and 1845 cm^-1^ for EPI, EPIIS, ISOP, and PILO, respectively. A deformation in the dihydrofuran ring represented the second highest intensity, at a frequency between 1075 cm^-1^ and 1162 cm^-1^ in all alkaloids. This was followed by C-N stretching and C-H group deformation, which occurred at frequencies between 1404 cm^-1^ and 1455 cm^-1^, respectively, in all alkaloids. The OH group showed a strong peak in the region of 3783 cm^-1^ to 3830 cm^-1^. This peak was absent in MAC, because it does not contain an OH group in its structure (S8 Table). The lowest values of the IR frequencies were identified in PILO, which corroborated the results presented in Fig 2 that demonstrated a lower stability for this alkaloid (S8 Table). For the IR and Raman parameters, the theoretical model B3lyp/6-31+G(d,p) was highly similar to the B3lyp/6-311++G(d,p), and the B3lyp/Sdd model indicated a more distant wavenumber than these two models (S3 and S4 Figs).

The experimental IR data for the EPI alkaloid have been previously described [19], including C = O stretching in dihydrofuran (1769 cm^-1^), C-C stretching in imidazole (1568 cm^-1^), symmetric stretching N-C-N in imidazole (1524 cm^-1^), and C-C stretching in benzene (1472 cm^-1^), which showed a greater similarity to our theoretical parameters compared with those used by the author, which supported the use of the same theoretical model. This difference might have resulted from the optimization of the chemical structure of EPI used differently in both works.

The theoretical ^13^C NMR spectra of the EPI, EPIIS, ISOP, PILO, and MAC alkaloids are shown in S5 Fig, as well as the chemical shift patterns (S6 Table). The ^13^C NMR spectrum of EPI shows the peaks related to the CH_3_ and CH_2_ groups (C2 and C4) at 33–37 ppm and CH (C8) at 85 ppm, where the latter exhibits a hydroxyl group, unlike C4. The C1 and C3 (143–151 ppm) of the imidazole ring were displaced, owing to the resonance of the aromatic ring. In addition, the resonance peaks at 131–134 ppm related to CH bonds (C10, C11, C12, C13, and C14) in benzene were observed to be slightly less displaced than the C1 and C3 of the imidazole aromatic ring. Finally, a downfield shift for C6 in the dihydrofuran ring was observed, which was displaced by the binding to the oxygen atom, whereas the CH_2_ (C15) directly bound to the CO (carbonyl group) and C7 (CH) were protected by the ring. As the EPI, EPIIS, ISOP and PILO alkaloids exhibit isomerism, similarities were observed in the values of the displacement patterns (S5 Fig; S6 Table).

The experimental results of ^13^C NMR of EPI, EPIIS, ISOP, PILO, and MAC presented in previous studies [16, 19] describe patterns of displacement similar to that obtained in our calculations, with peaks of (179, 137, 32, 125–128 ppm) for EPI, (180, 139, 31, 126–129 ppm) for EPIIS, (179, 139, 31, 127–129 ppm) for ISOP, (179, 139, 31, 127–129 ppm) for PILO, and (178, 135, and 33 ppm) for MAC.

#### Electronic density

The dipole moment calculations presented in the three theoretical models showed closer values between the B3lyp/6-311++G(d,p) and B3lyp/6-31+G(d,p) models. The data generated for all alkaloids using the B3lyp/6-311++G(d,p) model did result in significant differences because of the markedly close values of parameters; differences in the house of 0.2 debye, PILO (7.3 debye), EPI (5.8 debye), EPIIS (5.7 debye), ISOP (5,3 debye), and MAC (4.5 debye) are presented in S7 Table. These values also suggest a similar solubility potential.

**Fig 6:**
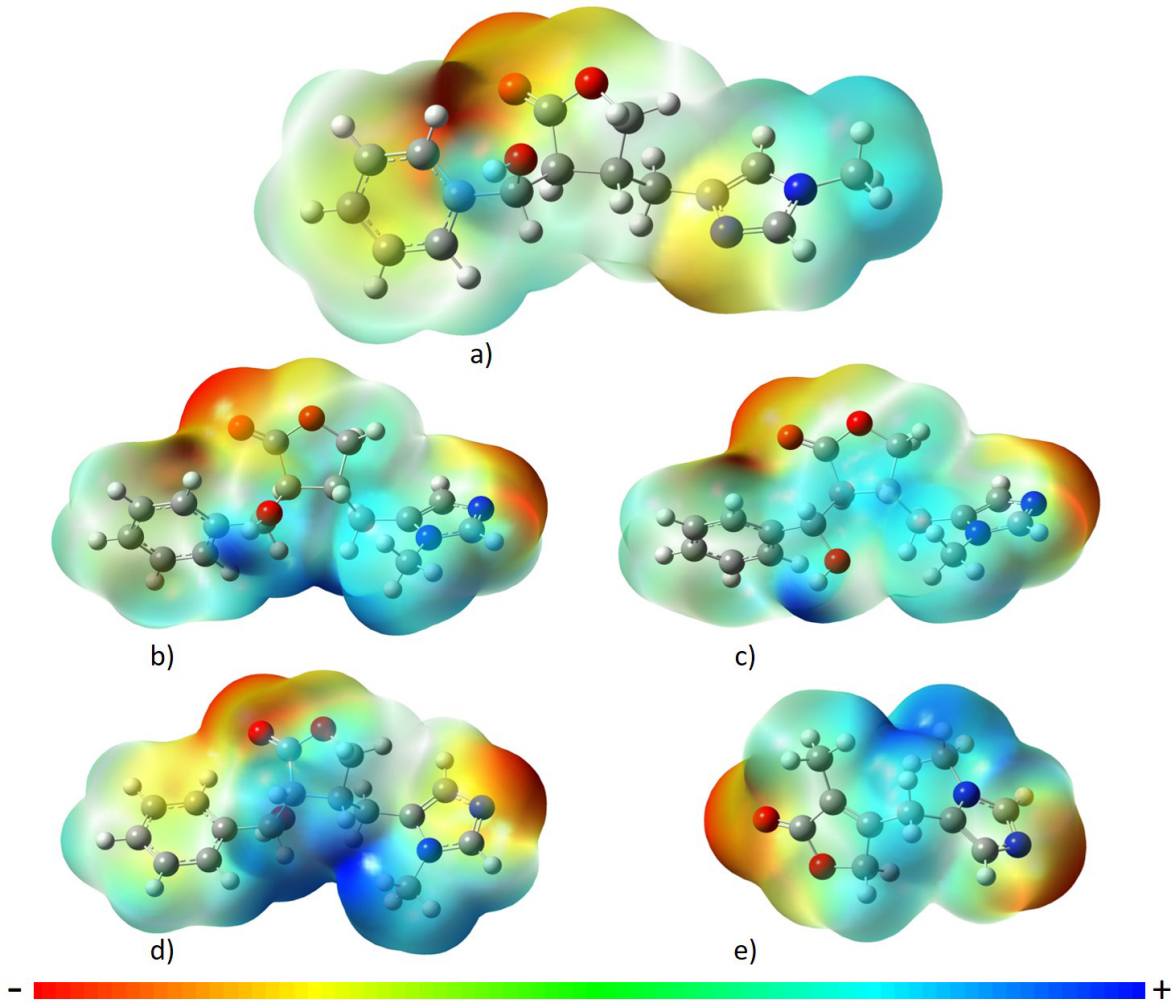
Electronic density of alkaloids. a) epiisopiloturine, b) epiisopilosine, c) isopilosine, d) pilosine, and e) macaubine, using the theoretical model B3lyp/6-311++G(d,p). The colors represent red (negative) and blue (positive).

For the electron density in each molecule, it was observed that redder regions are more negative and therefore more polar; this was observed in the imidazolic ring by the N1 atom and in the O = C group of the dihydrofuran ring in all the alkaloids (Fig 6). These regions with higher electronic density also represented sites with a higher probability of chemical interactions, described in molecular docking.

### ADMET predictions

The theoretical prediction of pharmacokinetics in sílico is an approach that is currently used widely in the initial study of the ADMET properties to reduce unnecessary expense in biological assays of compounds with a high probability of pharmacokinetic problems, which saves on time and investment [50].

ADMET analysis for the alkaloids produced the same values for the EPIIS, ISOP and PILO optical isomers, which assigned all three the same prediction in Table 4. Through analysis of the plasma protein binding property (PPB), similar values were found for the EPI, EPIIS, ISOP, and PILO isomers (63%), with a lower value found for the MAC alkaloid (37%). The results of the penetration potential in the blood–brain barrier (BBB) showed that the compounds have low potential to cross the blood–brain barrier, indicated by values below 0.1 [51]; the obtained values were 0.011 (EPI), 0.016 (EPIIS), and 0.992 (MAC), indicating that these compounds were less likely to cause side effects in the central nervous system [52].

**Table 4:** ADMET predictions of the epiisopiloturine, epiisopilosine, isopilosine, pilosine and macaubine alkaloids.

ADMET	EPI	EPIIS/ISOP/PILO	MAC
Plasma protein binding (PPB) (%)	63.140757	63.940875	37.146972
Blood-brain barrier penetration (BBB) (C, brain/C, blood)	0.011675	0.0167958	0.992212
Skin Permeability (logKp, cm/h)	-3.86369	-3.89764	-3.0451
Human intestinal absorption (HIA, %)	96.050121	96.050121	97.523105
Caco-2 cell Permeability (nm/s)	21.8393	22.8681	27.2895
MDCK cell permeability (nm/s)	16.0498	9.63097	15.7929
P-glycoprotein inhibition	Non	Non	Non
Water solubility in buffer (mg/L)	23332.8	78158.6	3810.15
Pure water solubility (mg/L)	8525.04	21880	110732
Ames test	Mutagen	Mutagen	Mutagen
Ames TA100 (+S9)	Negative	Negative	Positive
Ames TA100 (-S9)	Negative	Negative	Positive
Ames TA1535 (+S9)	Negative	Negative	Positive
Ames TA1535 (-S9)	Negative	Negative	Positive
Carcinogenicity (Mouse)	Negative	Negative	Negative
Carcinogenicity (Rat)	Negative	Negative	Negative
CYP 2C19 inhibition	Inhibitor	Inhibitor	Inhibitor
CYP 2C9 inhibition	Inhibitor	Inhibitor	Inhibitor
CYP 2D6 inhibition	Non	Non	Non
CYP 2D6 substrate	Non	Non	Weakly
CYP 3A4 inhibition	Inhibitor	Inhibitor	Inhibitor
CYP 3A4 substrate	Substrate	Substrate	Substrate
Lipinski’s Rule	Suitable	Suitable	Suitable
WDI-like Rule	Within 90% cutoff	Within 90% cutoff	Within 90% cutoff
Lead-like Rule	Binding affinity > 0.1 μM	Binding affinity > 0.1 μM	Violated
CMC-like Rule	Qualified	Qualified	Qualified
MDDR-like Rule	Mid-structure	Mid-structure	Mid-structure

The values the permeability through human skin were −3.863 cm/h (EPI), −3.897 cm/h (EPIIS), and −3.045 cm/h (MAC); therefore, the compounds cannot be absorbed through human skin [53]. In the analysis of human intestinal absorption (HIA), one of the main parameters for new drug candidates, the analyzed alkaloids had values of 96.0% (EPI and EPIIS) and 97.5% (MAC). Some studies [54–55] have shown that values between 70% and 100% indicate good intestinal absorption.

The recommended parameters for the prediction of oral absorption of drugs use two permeability models, Caco-2 and MDCK cells. The values obtained for the alkaloids were considered intermediate [56]: 27.28 nm/s for MAC, 22.86 nm/s for EPIIS, and 21.83 nm/s for EPI in Caco-2 cells; and 15.79 nm/s for MAC, 16.04 nm/s for EPI, and 9.63 nm/s for EPIIS in MDCK cells.

None of the alkaloids inhibited P-glycoprotein, a protein responsible for the absorption, distribution, metabolism, and excretion of several different drugs [57]. In relation to solubility in water and pure water, the alkaloids EPIIS, ISOP, and PILO achieved higher values of 78,158.6 mg/L and 21,880 mg/L, respectively.

Data on the interaction with cytochrome P450 (CYP) protein indicated that all alkaloids were inhibitors of CYP 2C19, CYP 2C9, and CYP 3A4; this reduces the ability of these proteins to metabolize other drugs in the body, which suggests the possible accumulation of their potentiating metabolites to improve the pharmacological effect of these drugs. None of the alkaloids were found to be inhibitors of the CYP AD6 protein.

The characterization of mutagenicity by the Ames test indicated mutagenicity data for all tested alkaloids. However, in specific testing using TA100 and TA1535 cells, the results among the isomers were negative [58], and the alkaloids did not produce carcinogenicity in rats or mice.

All the alkaloids were suitable for drug classification by Lipinski’s rule (rule of five) and by the World Drug Index (WDI) as having a greater than 90% probability of solubility and permeability [59]. The Lipinski rule also has been applied [60] to evaluate ligands used in the formation of complexes with PNP. The isomeric alkaloids were classified as having higher binding affinity at 0.1 μM by the lead-like rule, except for MAC, which violated the rule [61]. In the CMC-like rule, all alkaloids were classified as qualified [62] and, for the MDDR-like rule, as an intermediate between a potential and non-potential drug [63].

As poor pharmacokinetic properties are important causes of late-stage failure in drug development, the reduction of these failures through the use of silicon tools such as ADME can lead to early predictions from the optimization of these properties [64].

### Molecular docking

The results of molecular docking of the alkaloids EPI, EPIIS, ISOP, PILO, and MAC with the enzymes of the *S*. *mansoni* worm are shown in Table 5.

**Table 5:** Molecular affinity parameters of the epiisopiloturine, epiisopilosine, isopilosine, pilosine and macaubine alkaloids with *S*. *mansoni* enzymes by the autodock program.

Complex (Protein-ligand)	ΔG_bind_ ^a^ (kcal mol^-1^)	Ki ^b^ (μM)	Number of independent docking runs	Number of conformations in the first ranked cluster	Amino acids that interact through hydrogen bonds ^c^	Amino acids that make hydrophobic interactions ^c^
Up/epiis	-7.68	2.36 μM	100	73	Arg203(2), Gln201, Met231	Arg121, Gly126, Glu234, Glu232, Ile265, Met233, Phe197, Phe272, Ser125, Thr124-10
Tgr/epiis	-7.46	3.4 μM	100	11	Glu300, Lys162	Ala470, Cys159, Leu441, Phe324, Phe474, Pro443, Thr471, Thr472, Tyr296, Val473-10
Tgr/isop	-7.37	3.97 μM	100	22	Glu300, Lys162(2), Thr472	Ala470, Cys159, Leu441, Phe324, Phe474, Pro443, Thr471, Tyr296, Val473-9
Tgr/epi	-7.25	4.89 μM	100	27	Lys162(2), Thr442	Cys159, Gly158, Glu300, Leu441, Phe280, Phe474, Pro443, Thr472, Tyr296, Val157, Val297, Val473-12
Up/isop	-7.19	5.37 μM	100	64	Arg50 (2), Arg121, Gln201, Thr124 (2)	Arg121, Gly123, Glu232, Met93, Met231, Met233, Phe197, Phe272-8
Pnp/epi	-7.18	5.46 μM	100	40	Ala118(2), His88	Asn117, Asn245, Ala119, Gly120, Gly220, Glu203, His259, Met221, Pro200, Tyr90, Tyr202, Val219–12
Up/epi	-7.16	5.68 μM	100	33	Arg50, Arg121(2), Thr124, Glu234, Gly46	Gly92, Gly123, Glu232, His91, Met93, Met231, Phe197, Phe272, Ser125–9
Tgr/pilo	-7.13	5.95 μM	100	20	Lys162, Thr442	Ala470, Cys159, Glu300, Leu441, Phe474, Pro443, Thr471, Thr472, Tyr296-9
Pnp/epiis	-7.11	6.19 μM	100	75	Ala118(2), Met221, Ser222	Asn117, Asn245, Gly120, Gly220, His88, His259, Tyr90, Tyr202, Thr244, Val219-10
Pnp/pilo	-7.08	6.47 μM	100	41	Ala118, Arg86, Met221	Ala119, Asn245, Gly34, Gly120, Gly220, Glu203, His88, Ser222, Tyr90, Tyr202, Val219–11
Mtap/isop	-6.91	8.55 μM	100	8	Ala88, Asn205, Met206	Ile182, Val204, Ser12, Phe187, Asp232, Gly90, Asp230, Thr229, Cys89, Thr207, His55
Pnp/isop	-6.83	9.84 μM	100	8	Ala118, Met221	Asn117, Asn245, Ala119, Arg86, Gly34, Gly120, Gly220, Glu203, His88, Ser35, Ser222, Tyr90, Tyr202, Val219-14
Up/pilo	-6.74	11.54 μM	100	99	Arg203 (2), Gln201, Met231	Arg121, Gly126, Glu232, Glu234, Ile265, Met233, Phe197, Phe272, Ser125, Thr124-10
Arg/pilo	-6.64	13.47 μM	100	1	Asn169, His171(2), Gly157	Glu307, Thr276, Asp158, Asp262, Asp264, His156, Asp211, Glu216, Asn160, Asp213, Gly172-11
Mtap/epiis	-6.63	13.72 μM	100	1	Asp230, Ser188	Ala88, Asn205, ILe182, Val204, Phe187, Met206, Gly90, Thr229, Cys89, Asp232
Mtap/epi	-6.54	15.97 μM	100	33	Ala88, His55, Met206, Ser12	Thr229, Pro63, Phe187, Asp320, Cys89, Val204, Gly90, Thr207, Asn205
2cb1/epiis	-6.52	16.65 μM	100	2	Gln94, Gly144, Gly269, His270	Cys100, Gly98, Gly143, Leu252, Leu267, Ser99, Trp101, Trp292, Val247
Mtap/pilo	-6.51	16.97 μM	100	84	Ala88, Asn205, Met206, Val204	Thr229, Cys89, Ile182, Phe187, Asp232, Gly90, Asp230
Arg/epi	-6.48	17.71 μM	100	54	Ala166, Ser165	Met173, Asn169, Gly172, His171, Asp158, Asp262, Asp264, Thr276, Asn160, Asp213, Ser167-11
Arg/epiis	-6.44	19.07 μM	100	16	Asn169 (2)	Gly172, Ser167, His171, Thr276, Gly157, His156, Glu216, Asp 158, Asp213, Asn160, Asp211, Ser165, Ala166-13
Hdac8/epi	-6.43	19.44 μM	100	54	-	Tyr99, Asp100, Phe151, Pro19, Tyr153, Lys20, Phe21, Pro102, Phe104, Tyr110, Ser18
Arg/isop	-6.4	20.34 μM	100	72	Ala166, Asp158, Ser165, Ser167	Asp213, Glu216, Asp211, His156, Gly157, His171, Met173, Asn160, Gly172, Asn169-10
2cb1/isop	-6.23	27.3 μM	100	8	Gln94, Gly269, Cys100, His270, Gly269	Gly98, Gly143, Gly144, Gly268, His181, Leu252, Leu267, Trp292, Val247
2cb1/pilo	-6.18	29.35 μM	100	10	Gly144(2), Gln94	Ala271, Cys100, Gly98, Gly143, Gly244, Gly269, Glu316, His270, Leu146, Trp101
Up/mac	-6.15	31.19 μM	100	97	Arg50, Gly46, Thr124	Arg121, Gly92, Gly123, Gln201, Glu232, Glu234, His91, Met93, Met233, Phe197-10
Hdac8/epiis	-6.14	31.78 μM	100	7	His141, His292,	Asp184, His142, Gly338, Asp186, Phe216, Gly150, Asp100, Asp285, Typ140, Phe21, Tyr341, His188, Phe151, Asp290
2cb1/epi	-6.09	34.36 μM	100	10	Gly144(2), Trp101	Ala271, Cys100, Gln94, Gly98, Gly143, Gly269, Glu316, His270, Leu146
2cb1/mac	-6.09	34.09 μM	100	2	Ala127, Phe175	Asp93, Arg92, Gly161, Glu124, Glu165, Lys164, Lys177, Ser126, Ser162, Ser163, Phe103, Tyr173
Arg/mac	-5.6	78.59 μM	100	100	Asp158, Gly157	Ser165, Asn169, Asn160, Ser167, Gly172, Ala166, His156, Glu216, Asp211-9
Mtap/mac	-5.54	87.42 μM	100	100	Ser188	Thr229, Cys89, Gly90, Asp230, Asp232, Phe187, Ala88, Asn205, Val204, Met206, Ile182
Hdac8/pilo	-5.54	87.15 μM	100	2	Tyr99	Phe104, Pro102, Tyr153, Asp100, Ser18, Pro19, Lys20, Phe151
Hdac8/isop	-5.41	108.27 μM	100	14	Asp186, Asp285, His188, Phe216	His292, Phe215, Asp100, Gly150, Tyr341, Phe151, His142
Hdac8/mac	-5.38	113.26 μM	100	61	-	Asp100, Pro102, Tyr153, Phe104, Tyr110, Lys20, Tyr99, Phe151, Ser18, Pro19
Tgr/mac	-5.36	117.12 μM	100	1	Cys154, Thr153	Ala256, Ala445, Asp433, Gly118, Gly258, Gly432, Ile431, Ile434, Leu441, Ser117, Thr442-11
Pnp/mac	-5.36	118.65 μM	100	13	Asn245, Tyr90	Asn117, Ala118, Ala119, Gly120, Gly220, His88, Met221, Ser222, Tyr194, Tyr202, Thr244, Val219, Val262-13

^a^ Binding energy of the best conformation

^b^ Inhibition constant of the best conformation

^c^ Obtained using Ligplot+ software

**Fig 7:**
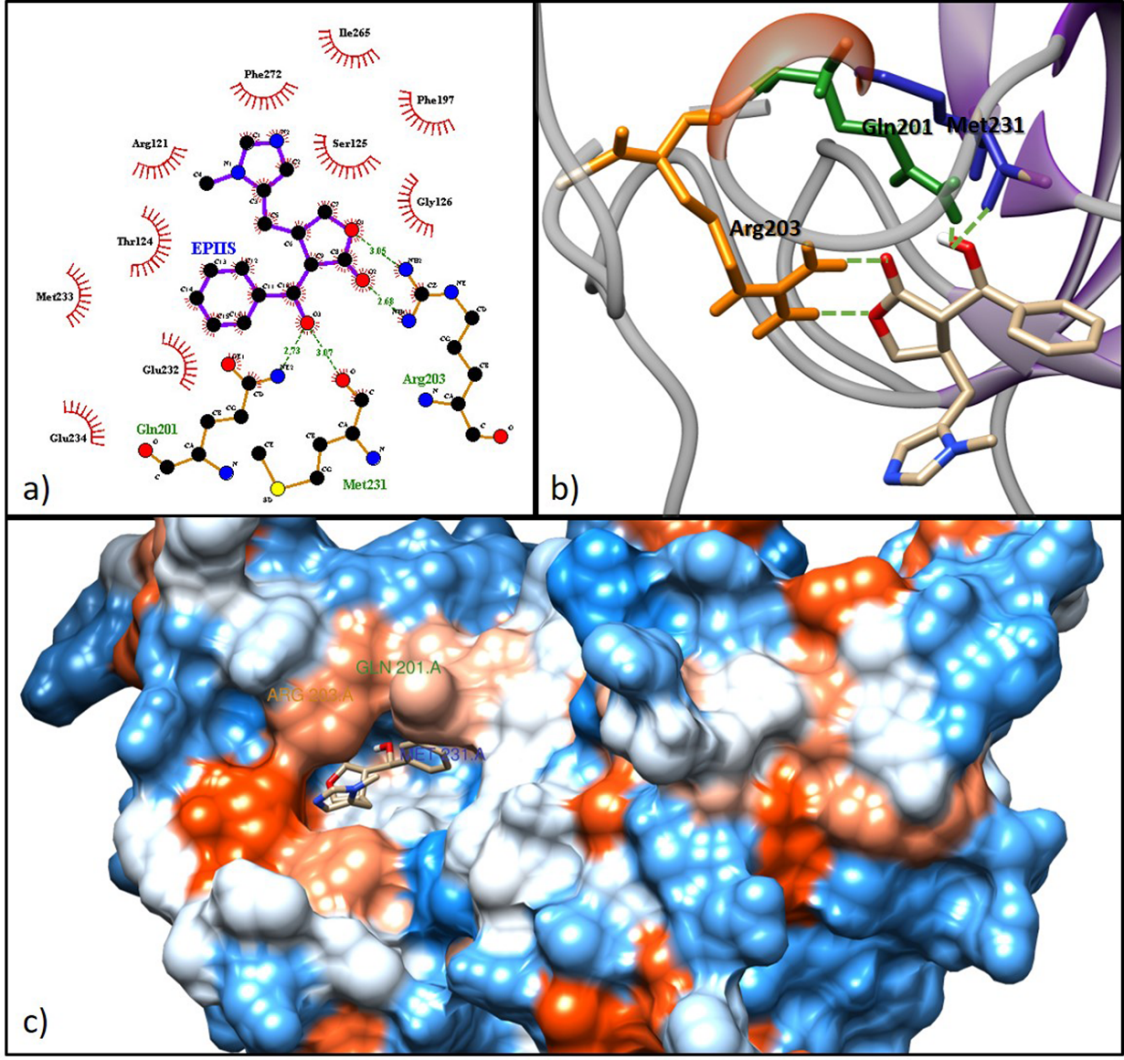
Molecular docking of the epiisipilosine alkaloid with the UP enzyme of *S*. *mansoni*. a) 2D scheme showing the hydrogen bonds and hydrophobic interactions in the EPIIS-UP complex. b) 3D interactions by hydrogen bonds (Gln201, Met231 and Arg203) in EPIIS. c) 3D conformation of the active site of EPIIS binding in UP enzyme.

The enzyme putative uridine phosphorylase (UP) and the EPIIS ligand demonstrated the highest molecular affinity, with a binding energy of −7.68 kcal mol^-1^ and an inhibition constant of 2.36 μM. UP/epiis complex formed hydrogen bonds with three amino acid residues (Arg203, Gln201, and Met231) (Fig 7). It was also observed that all ligands that interacted with this enzyme had hydrogen bonds or hydrophobic interactions with the amino acid Gln201 in the active site of the protein. The UP protein is a nucleoside phosphorylase that catalyzes the N-ribosidic binding of uridine and thymidine to produce ribose-1-phosphate, uracil, and thymine. This enzyme has important metabolic roles, including protection against ischemia, lipid metabolism, and protein acetylation [65].

**Fig 8:**
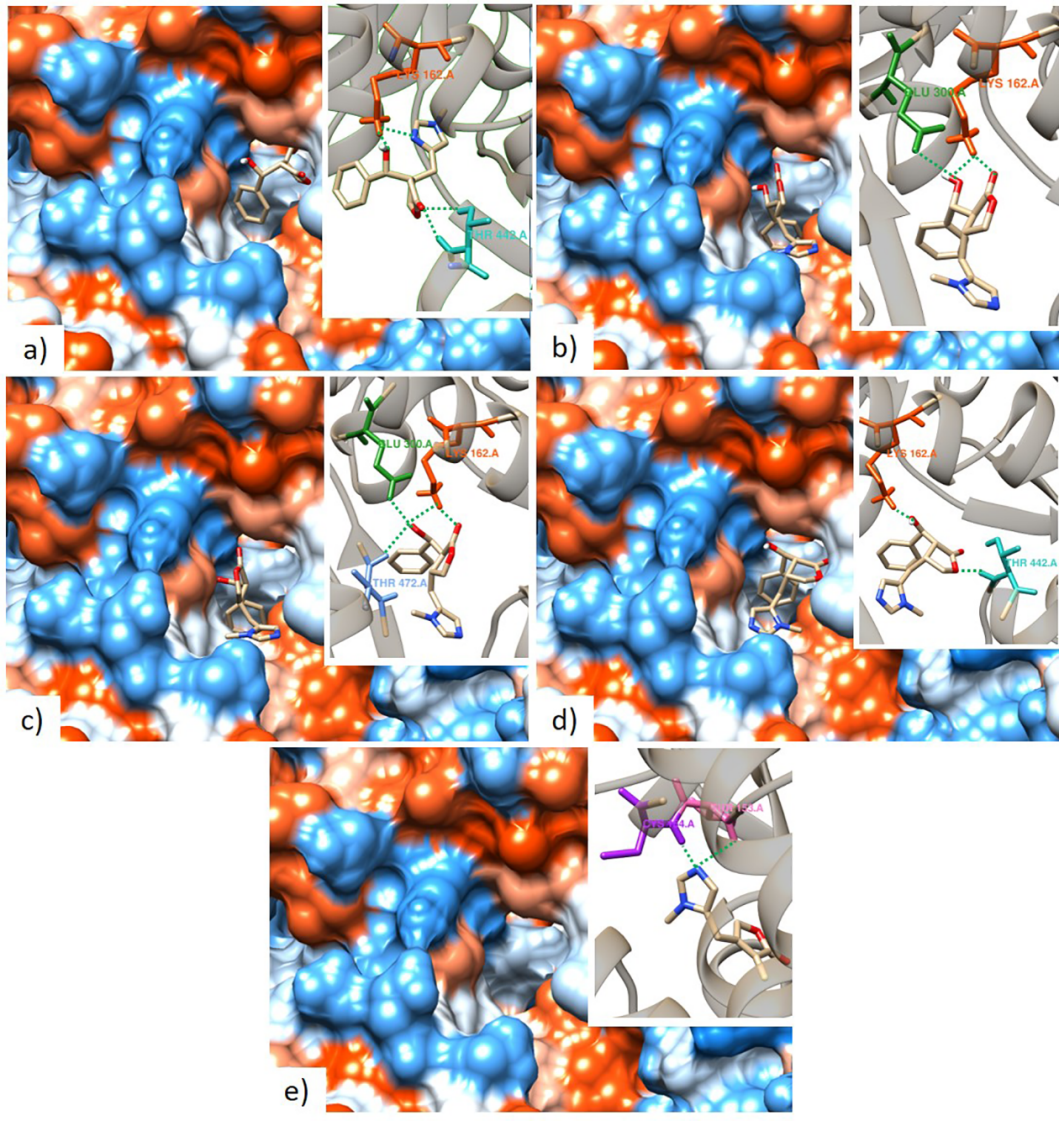
Molecular docking showing the active site of the TGR enzyme with the alkaloids and their interactions by hydrogen bonds. a) epiisopiloturine, b) epiisopilosine, c) isopilosine, d) pilosine, and e) macaubina.

The interaction of the enzyme thioredoxin glutathione reductase (TGR) with the EPIIS ligand showed a binding energy of −7.46 kcal mol^-1^ and an inhibition constant of 3.4 μM. In this complex, only three hydrogen bonds were formed, at the Glu300 (O3) and Lys162 (O2 and O3) residues (Table 5; Fig 8). The ISOP alkaloid has been shown to also interact with the amino acids Glu300 and Lys162 of EPIIS, in addition to the Thr472 residue, although this was not sufficient to produce higher energy than EPIIS. In the same TGR enzyme, the EPI and PILO alkaloids also interact with the amino acids Lys162 and Thr442, but the position in the active site of the enzyme gave EPI the formation of four hydrogen bonds, which may have offered higher affinity energy for this ligand (Fig 8). The amino acid of the active site Tyr296 of this enzyme interacted with all ligands, except for MAC (Fig 8). This same Tyr296 residue was also identified in other studies of the interaction of the TGR enzyme with anti-schistosomal molecules [52]. TGR is a chimeric flavoenzyme related to detoxification and parasite survival in the host organism [66]; in addition to this, the enzyme participates directly in parasite homeostasis, where it acts as a detoxificant of the reactive oxygen species (ROS) present in the blood vessels of the definitive host and allowing the survival of the worm [64].

The enzyme purine nucleoside phosphorylase (PNP) showed a higher binding affinity with the EPI and EPIIS ligands, with binding energies of −7.18 kcal mol^-1^ and −7.11 kcal mol^-1^, respectively, and inhibition constants of 5.46 μM and 6.19 μM, respectively. In all ligands, it was possible to observe interactions with the amino acid Ala118 at the active site by hydrogen bonds or hydrophobic interaction. This Ala118 amino acid has also been identified in the literature [60] as an important residue in the testing of *S*. *mansoni* inhibitors against PNP. In addition, the same authors observed interactions with Met221 and His259 residues, which were also observed to interact with EPIIS and EPI (Table 5). The PNP enzyme of *S*. *mansoni* is essential for the recovery of purine and nucleoside bases in schistosomes; it catalyzes reversible purine phosphorylase (2'-deoxy) ribonucleosides in the presence of inorganic orthophosphate (Pi) as a second substrate to yield the corresponding purine and (2'-deoxy) ribose-1-phosphate base as products [60,67].

The enzyme methylthioadenosine phosphorylase (MTAP) showed higher affinity to ISOP (−6.91 kcal mol^-1^; 8.55 μM) and EPIIS (−6.63 mol^-1^; 13.72 μM). All ligands were shown to have contact with the amino acid Asp230 at the active site of the MTAP enzyme (Table 5). This enzyme uses adenosine as a substrate for the production of adenine. As it is likely the only route for purine production, it represents a promising target for therapies against schistosomiasis [68].

The affinity parameters for the enzyme arginase (ARG) showed stronger interactions with PILO (−6.64 kcal mol^-1^, 13.47 μM) and EPI (−6.48 kcal mol^-1^, 17.71 μM). It was also observed that all ligands had hydrogen bond or hydrophobic contact with the amino acid Asp158 of the active site of this enzyme (Table 5). ARG is a binuclear holoenzyme that catalyzes the hydrolysis of L-arginine to L-ornithine and urea, which affects the biosynthesis processes of NO. It regulates all forms of the parasite that interact with the human host, and this enzyme is believed to play a role in the parasitic immune response [69].

The formation of an enzyme complex with cathepsin B1 (2CB1) and the EPIIS ligand showed higher affinity with a binding energy of −6.52 kcal mol^-1^ and an inhibition constant of 16.65 μM. The protein-ligand complex showed four hydrogen bonds (Gln94, Gly144, Gly269, and His270) and it was possible to observe the contact of the active site Cys100 amino acid in all ligands except MAC (Table 5). The Cys100 and His270 residues were also observed in the active site of smCB1 complexed with heparin [70]. Adult *S*. *mansoni* living in the human cardiovascular system require nutrients from the blood for their growth, development, and reproduction, and smCB1 has been established a protease associated with the intestines of worms that digests host blood proteins as a source of nutrients [70].

The histone deacetylase 8 (HDAC8) enzyme demonstrated better affinity parameters with EPI (−6.43 kcal mol^-1^, 19.44 μM) and EPIIS (−6.14 kcal mol^-1^, 31.78 μM). Although EPI binding was higher for this enzyme, it was not possible to verify the presence of hydrogen bonds, only of hydrophobic bonds. The Tyr341 residue of the active site of the HDAC8 enzyme was observed to interact with the EPI and EPIIS linkers (Table 5). Other studies [71] have also identified the Tyr341, His292, and His141 residues present in this complex in addition to the importance of the zinc ion. *S*. *mansoni* HDAC8 is an acetyl-L-lysine deacetylase that plays a key role in parasite infectivity, controlling post-transcriptional acetylation and deacetylation in the DNA and gene regulation. These enzymes are linked to potential anticancer, antiviral, antiparasitic, and anti-inflammatory targets [71–72].

The data presented in the literature for the five alkaloids corroborate with published experimental studies in which the in vitro activity of the EPIIS alkaloid against *S*. *mansoni* was 3.125 μg/mL [16], followed by EPI (300 μg/mL) [20], ISOP (500 μg/mL) [16], and MAC, which showed no activity up to a concentration of 500 μg/mL [16].

## Conclusions

The molecular geometries; electronic structures; quantum chemical descriptors; and infrared, Raman, RMN, and UV-Visible spectra of the EPI, EPIIS, ISOP, PILO, and MAC alkaloids have been studied by DFT calculation. The theoretical model B3lyp/6-311++G(d,p) was the most satisfactory to describe all the studied properties, and can be considered suitable for use as a reference for similar structures that showed this pharmacological activity. This work provides new insight to the potential of these molecules as drugs against schistosomiasis, through the identification of previously unknown properties, expansion of the field of study, and enabling the discovery of novel treatments for this disease.

The ADMET profile showed that the EPI alkaloid and its isomers are suitable for the standard Lipinski classification of drugs, with positive parameters in PPB, BBB, HIA and CYP inhibitors, besides having low toxicity and carcinogenicity with more than 90% probability of having good solubility and permeability.

Molecular docking evidenced that the EPIIS alkaloid was the ligand that presented better affinity parameter for UP and TGR proteins, indicating a possible drug candidate for this disease.

## Supporting information

S1 Table Atomic charges by the Mulliken, Chelpg, and NBO methods of the epiisopiloturine, epiisopilosine, isopilosine, pilosine and macaubine alkaloids using the theoretical model B3lyp/Sdd. (DOCX)

S2 Table Atomic charges by the Mulliken, Chelpg, and NBO methods of the epiisopiloturine, epiisopilosine, isopilosine, pilosine and macaubine alkaloids using the theoretical model B3lyp/6-31+G(d,p). (DOCX)

S3 Table Distances of the atomic bonds and atomic angles of the epiisopiloturine, epiisopilosine, isopilosine, pilosine and macaubine alkaloids using the theoretical models B3lyp/Sdd and B3lyp/6-31+G(d,p). (DOCX)

S4 Table Electrostatic energies in (Hartree) of epiisopiloturine, epiisopilosine, isopilosine, pilosine and macaubine alkaloids using the theoretical models B3lyp/Sdd, B3lyp/6-31+G(d,p) and B3lyp/6-311++G(d,p). Gibbs^a^, Enthalpy^b^, Thermal^c^, Energy zero point^d^.
(DOCX)

S5 Table Molecular orbital values HOMO and LUMO (in eV) of epiisopiloturine, epiisopilosine, isopilosine, pilosine and macaubine alkaloids using the theoretical models B3lyp/Sdd, B3lyp/6-31+G(d,p) and B3lyp/6-311++G(d,p). (DOCX)

S6 Table Epiisopiloturine, epiisopilosine, isopilosine, pilosine and macaubine ^13^C NMR chemical shifts. Atom labels accordingly to S5 Fig.
(DOCX)

S7 Table Dipole moment in epiisopiloturine, epiisopilosine, isopilosine, pilosine and macaubine alkaloids using the theoretical models B3lyp/Sdd, B3lyp/6-31+G(d,p), and B3lyp/6-311++G(d,p). (DOCX)

S8 Table Infrared spectroscopic frequencies (cm^-1^) of the epiisopiloturine, epiisopilosine, isopilosine, pilosine and macaubine alkaloids and their assignments using the theoretical model B3lyp/6-311++G(d,p). (DOCX)

S1 Fig UV-Vis spectra of epiisopiloturine, epiisopilosine, isopilosine, pilosine and macaubine alkaloids using the theoretical models B3lyp/Sdd and B3lyp/6-31+G(d,p). (TIF)

S2 Fig Infrared and Raman spectra of the epiisopiloturine, epiisopilosine, isopilosine, pilosine and macaubine alkaloids using the theoretical model B3lyp/6-311++G(d,p). a) IR spectra of the five alkaloids, b) zoom of the highest IR peak, c) Raman spectra of the five alkaloids, and d) zoom of the highest Raman peak.
(TIF)

S3 Fig Infrared spectra of epiisopiloturine, epiisopilosine, isopilosine, pilosine and macaubine alkaloids using the theoretical models B3lyp/Sdd, B3lyp/6-31+G(d,p), and B3lyp/6-311++G(d,p). (TIF)

S4 Fig Raman spectra of epiisopiloturine, epiisopilosine, isopilosine, pilosine and macaubine alkaloids using the theoretical models B3lyp/Sdd, B3lyp/6-31+G(d,p), and B3lyp/6-311++G(d,p). (TIF)

S5 Fig Epiisopiloturine, epiisopilosine, isopilosine, pilosine and macaubine ^13^C NMR chemical shifts using the theoretical model B3lyp/6-311++G(d,p). (TIF)

## References

[1] Schistosomiasis [Internet]. World Health Organization [cited 2017 Oct 10]. Available from: http://www.who.int/schistosomiasis.

[2] Moraes J. Natural products with antischistosomal activity. Future Med Chem. 2015;7: 801–820. doi: 10.4155/fmc.15.23 25996071 10.4155/fmc.15.23

[3] Lago EM, Xavier RP, Teixeira TR, Silva LM, Filho AAS, Moraes J. Antischistosomal agents: state of art and perspectives. Future Med Chem. 2017;10:89–120. doi: 10.4155/fmc-2017-0112 29235368 10.4155/fmc-2017-0112

[4] Mafud AC, Ferreira LG, Mascarenhas YP, Andricopulo AD, Moraes J. Discovery of novel antischistosomal agents by molecular modeling approaches. Trends Parasitol. 2016;32:874–886. doi: 10.1016/j.pt.2016.08.002 27593339 10.1016/j.pt.2016.08.002

[5] Ewing TJ, Makino S, Skillman AG, Kuntz I. D. DOCK 4.0: Search strategies for automated molecular docking of flexible molecule databases. J Comput Aid Mol Des. 2001;15:411–421. 10.1023/a:1011115820450 11394736

[6] Chen M, Zeng G, Xu P, Zhang Y, Jiang D, Zhou S. Understanding enzymatic degradation of single-walled carbon nanotubes triggered by functionalization using molecular dynamics simulation. Environ. Sci. Nano. 2017; 4:720–727.

[7] Chen M, Qin X, Zeng G. Biodegradation of Carbon Nanotubes, Graphene, and Their Derivatives. Trends Biotechnol. 2017; 35:836–846. doi: 10.1016/j.tibtech.2016.12.001 28063621 10.1016/j.tibtech.2016.12.001

[8] Chen M, Zeng G, Xu P, Yan M, Xiong W, Zhou S. Interaction of carbon nanotubes with microbial enzymes: conformational transitions and potential toxicity. Environ. Sci. Nano. 2017; 4:1954–1960.

[9] Chen M, Zeng G, Lai C, Zhang C, Xu P, Yan M, Xiong W. Interactions of carbon nanotubes and/or graphene with manganese peroxidase during biodegradation of endocrine disruptors and triclosan. Chemosphere. 2017; 184:127–136. doi: 10.1016/j.chemosphere.2017.05.162 28586653 10.1016/j.chemosphere.2017.05.162

[10] Coleman RG, Carchia M, Sterling T, Irwin JJ, Shoichet BK. Ligand Pose and Orientational Sampling in Molecular Docking. Plos One. 2013; 8:e75992 doi: 10.1371/journal.pone.0075992 24098414 10.1371/journal.pone.0075992 PMC3787967

[11] Chen H, Yao K, Nadas J, Bode AM, Malakhova M, Oi N, Li H, Lubet RA, Dong Z. Prediction of Molecular Targets of Cancer Preventing Flavonoid Compounds Using Computational Methods. Plos One. 2012; 7:e38261 doi: 10.1371/journal.pone.0038261 22693608 10.1371/journal.pone.0038261 PMC3365021

[12] Kerns E, Li D. Drug-like properties: concepts, structure design, and methods: from ADME to toxicity optimization. 1 ed, 514p; 2008.

[13] Kramer C, Ting A, Zheng H, Hert J, Schindler T, Stahl M, Robb G, Crawford JJ, Blaney J, Montague S, Leach AG, Dossetter AG, Griffen EJ. Learning Medicinal Chemistry Absorption, Distribution, Metabolism, Excretion, and Toxicity (ADMET) Rules from Cross-Company Matched Molecular Pairs Analysis (MMPA). J. Med. Chem. 2018; 61:3277–3292. doi: 10.1021/acs.jmedchem.7b00935 28956609 10.1021/acs.jmedchem.7b00935

[14] Guimarães MA, Campelo YD, Véras LM, Colhone MC, Lima DF, Ciancaglini P, Kuckelhaus SS, Lima FC, de Moraes J, de Leite JR. Nanopharmaceutical approach of epiisopiloturine alkaloid carried in liposome system: preparation and in vitro schistosomicidal activity. J Nanosci Nanotechnol. 2014;14:4519–4528. 24738423 10.1166/jnn.2014.8248

[15] de Lima LI, Py-Daniel KR, Guimarães MA, Muehlmann LA, Mafud AC, Mascarenhas YP, Moraes J, de Souza de Almeida Leite JR, Jiang CS, Azevedo RB, Figueiró Longo JP. Self-nanoemulsifying drug-delivery systems improve oral absorption and antischistosomal activity of epiisopiloturine. Nanomedicine (Lond). 2018; 10.2217/nnm-2017-0308. 10.2217/nnm-2017-0308 29564947

[16] Rocha JA, Andrade IM, Véras LMC, Quelemes PV, Lima DF, Soares MJS, et al Anthelmintic, antibacterial and cytotoxicity activity of imidazole alkaloids from *Pilocarpus microphyllus* leaves. Phytother Res. 2017;31:624–630. doi: 10.1002/ptr.5771 28111828 10.1002/ptr.5771

[17] Guimarães MA, De Oliveira RN, Véras LMC, Lima DF, Campelo YDM, Campos AS, et al Anthelmintic activity in vivo of epiisopiloturine against juvenile and adult worms of *Schistosoma mansoni*. PLoS Negl Trop Dis. 2015;9:e0003656 doi: 10.1371/journal.pntd.0003656 25816129 10.1371/journal.pntd.0003656 PMC4376696

[18] Voigtlander HW, Balsam G, Engelhardt M, Pohl L. Epiisopiloturin, ein neues pilocarpus-alkaloid. Archiv Pharmacal. 1978;311:927–935.

[19] Veras LMC, Cunha VRR, Lima FCDA, Guimarães MA, Vieira MM, Campelo YDM, et al Industrial scale isolation, structural and spectroscopic characterization of epiisopiloturine from *Pilocarpus microphyllus* Stapf leaves: a promising alkaloid against schistosomiasis. Plos One. 2013;8:e66702 doi: 10.1371/journal.pone.0066702 23840522 10.1371/journal.pone.0066702 PMC3694155

[20] Veras LM, Guimarães MA, Campelo YD, Vieira MM, Nascimento C, Lima DF, et al Activity of epiisopiloturine against *Schistosoma mansoni*. Curr Med Chem. 2012;19:2051–2058. 22420337 10.2174/092986712800167347

[21] Campelo YDM, Mafud A, Véras L, Guimarães MA, Yamaguchi L, Lima D, et al Synergistic effects of in vitro combinations of piplartine, epiisopiloturine and praziquantel against *Schistosoma mansoni*. Biomed Pharmacother. 2017;88:488–499. doi: 10.1016/j.biopha.2016.12.057 28126674 10.1016/j.biopha.2016.12.057

[22] Silva VG, Silva RO, Damasceno SRB, Carvalho NS, Prudêncio RS, Aragão KS, et al Anti-inflammatory and antinociceptive activity of epiisopiloturine, an imidazole alkaloid isolated from *Pilocarpus microphyllus*. J Nat Prod. 2013;76:1071–1077. doi: 10.1021/np400099m 23734744 10.1021/np400099m

[23] Nicolau LAD, Carvalho NS, Pacífico DM, Lucetti L T., Aragão K. S, Véras L. M. C, et al Epiisopiloturine hydrochloride, an imidazole alkaloid isolated from *Pilocarpus microphyllus* leaves, protects against naproxen-induced gastrointestinal damage in rats. Biomed Pharmacother. 2017;87:188–195. doi: 10.1016/j.biopha.2016.12.101 28056423 10.1016/j.biopha.2016.12.101

[24] Frisch MJ, Trucks GW, Schlegel HB, Scuseria GE, Robb MA, Cheeseman JR, et al Gaussian09, Revision C.01; Gaussian, Inc.: Wallingford, CT, 2010.

[25] Dennington RD, Keith TA, Millan JM. *GaussView*, v. 5.0.8, Semicchem, Inc.: Shawnee KS, 2008.

[26] Kohn W, Sham L. Self-consistent equations including exchange and correlation effects J Phys Rev. 1965;140:1133–1138.

[27] Lee C, Yang W, Parr RG. Development of the Colle-Salvetti correlation-energy formula into a functional of the electron-density. Phys Rev B. 1998;37:785–789. 10.1103/physrevb.37.785 9944570

[28] Becke AD. Density-functional thermochemistry. III. The role of exact exchange. J Chem Phys. 1993;98:5648–5652.

[29] Mclean AD, Chandler GS. Contracted Gaussian-basis sets for molecular calculations. 1. 2nd row atoms, Z = 11–18. J Chem Phys. 1980;72:5639–5648.

[30] Raghavachari K, Binkey JS, Seeger R, Pople JA. Self-consistent molecular orbital methods. 20. Basis set for correlated wave-functions. J Chem Phys. 1980;72:650–654.

[31] Parr RG, Von Szentpály L, Liu S. Electrophilicity index J Am Chem Soc. 1999;121:1922–1924.

[32] Zhan CG, Nichols JA, Dixon DA. Ionization potential, electron affinity, electronegativity, hardness, and electron excitation energy: molecular properties from density functional theory orbital energies. J Phys Chem A. 2003;107:4184−4195.

[33] Casida ME. Time-dependent density functional response theory for molecules In Chong DP, editor. Recent advances in density functional methods. Singapore: World Scientific; 1995.

[34] Stratmann RE, Scuseria GE, Frisch MJ. An efficient implementation of time-dependent density-functional theory for the calculation of excitation energies of large molecules. J Chem Phys. 1998;109:8218–8224.

[35] Gorelsky SI. Swizard. Toronto, ON: Department of Chemistry, York University; 1999.

[36] Berman HM, Westbrook J, Feng Z, Gilliland G, Bhat TN, Weissig H, et al The protein data bank. Nucleic Acid Res. 2000;28:235–242. 10592235 10.1093/nar/28.1.235 PMC102472

[37] Goodsell DS, Morris GM, Olson AJ. Automated docking of flexible ligands: applications of autodock. J Mol Recognit. 1996;9:1–5. doi: 10.1002/(SICI)1099-1352(199601)9:1&lt;1::AID-JMR241&gt;3.0.CO;2-6 8723313 10.1002/(sici)1099-1352(199601)9:1<1::aid-jmr241>3.0.co;2-6

[38] Goodsell DS. Computational docking of biomolecular complexes with Auto-Dock In: Golemis EA, Adams PD, editors. Protein-protein interactions: A molecular cloning mannual. 2nd ed New York: Cold Spring Harbor Laboratory Press; 2005.

[39] Morris GM, Huey R, Olson AJ. Using AutoDock for ligand-receptor docking In: Curr Protoc Bioinforma. Hoboken, NJ: John Wiley & Sons, Inc; 2008. 10.1002/0471250953.bi0814s24 19085980

[40] Sanner MF. Python: a programming language for software integration and development. J Mol Graph Model. 1999;17:57–61. 10660911

[41] Gasteiger J, Marsili M. Iterative partial equalization of orbital electro-negativity da rapid access to atomic charges. Tetrahedron. 1980;36:3219–3228.

[42] Morris GM, Goodsell DS, Halliday RS, Huey R, Hart WE, Belew RK, et al Automated docking using a Lamarckian genetic algorithm and an empirical binding free energy function. J Comput Chem. 1998;19:1639–1662.

[43] Solis FJ, Wets RJB. Minimization by random search techniques. Math Oper Res. 1981;6:19–30.

[44] Ramos RM, Perez JM, Baptista LA, De Amorim HL. Interaction of wild type, G68R and L125M isoforms of the arylamine-N-acetyltransferase from *Mycobaerium tuberculosis* with isoniazid: a computational study on a new possible mechanism of resistance. J Mol Model. 2012;18:4013–4024. doi: 10.1007/s00894-012-1383-6 22460521 10.1007/s00894-012-1383-6

[45] Atkins P, Jones L. Princípios de química: questionando a vida moderna e o meio ambiente. Porto Alegre: Bookman; 2012.

[46] Da Silva RR, Ramalho TC, Santos JM, Figueroa-Villar JD. On the limits of highest-occupied molecular orbital driven reactions: the frontier effective-for-reaction molecular orbital concept. J Phys Chem. 2006;110:1031–1040. 10.1021/jp054434y 16420004

[47] Maltarollo VG, Silva DC, Honório KM. Advanced QSAR studies on PPARδ ligands related to metabolic diseases. J. Braz. Chem. Soc. 2012;85–95.

[48] Pang X, Zhou L, Zhang M, Zhang L, Xu L, Xie F, Yu L, Zhang X. Two Rules on the Protein-Ligand Interaction. Open Conf Proc J. 2012; 3:70–80.

[49] Lu X, Wu CL, Wei S, Guo W. DFT/TD-DFT investigation of electronic structures and spectra properties of cu-based dye sensitizers. J Phys Chem A. 2010;114:1178–1184. doi: 10.1021/jp909731t 20000483 10.1021/jp909731t

[50] Kadan RU, Roy N. Recent trends in drug likeness prediction: a comprehensive review of in silico methods. Indian J Pharm Sci. 2007;69:609–615.

[51] Ma XL, Chen C, Yang J. Predictive model of blood-brain barrier penetration of organic compounds. Acta Pharm Sinic. 2005;26:500–512. 10.1111/j.1745-7254.2005.00068.x 15780201

[52] Mafud AC, Silva MPN, Nunes GBL, de Oliveira MAR, Batista LF, Rubio TI, Mengarda AC, Lago EM, Xavier RP, Gutierrez SJC, Pinto PLS, da Silva Filho AA, Mascarenhas YP, de Moraes J. Antiparasitic, structural, pharmacokinetic, and toxicological properties of riparin derivatives. Toxicol In Vitro. 2018;50:1–10. doi: 10.1016/j.tiv.2018.02.012 29476885 10.1016/j.tiv.2018.02.012

[53] Singh S, Singh J. Transdermal drug delivery by passive diffusion and iontophoresis: a review. Med Res Rev. 1993;13:569–621. 8412408 10.1002/med.2610130504

[54] Zhao YH, Le J, Abraham MH, Hersey A, Eddershaw PJ, Luscombe CN, et al Evaluation of human intestinal absorption data and subsequent derivation of a quantitative structure-activity relationship (QSAR) with the Abraham descriptors. J Pharm Sci. 2001;90:749–784. 11357178 10.1002/jps.1031

[55] Yee S. In vitro permeability across Caco-2 cells (colonic) can predict in vivo (small intestinal) absorption in man-fact or myth. Pharm Res. 1997;14:763–766. 9210194 10.1023/a:1012102522787

[56] Yamashita S, Furubayashi T, Kataoka M, Sakane T, Sezaki H, Tokuda H. Optimized conditions for prediction of intestinal drug permeability using Caco-2 cells. Eur J Pharm Sci. 2000;10:195–204. 10767597 10.1016/s0928-0987(00)00076-2

[57] Azeredo FJ, Uchôa FT, Costa TD. P-glycoprotein role on drug pharmacokinetics and interactions. Rev Bras Farm. 2009;90:321–326.

[58] Ames BN, Gurney EG, Miller JA, Bartsch H. Carcinogens as frameshift mutagens: metabolites and derivatives of 2-acetylaminofluorene and other aromatic amine carcinogens. Proc Nat Acad Sci. 1972;69:3128–3132. 4564203 10.1073/pnas.69.11.3128 PMC389719

[59] Lipinski CA, Lombardo F, Dominy BW, Feeney PJ. Experimental and computational approaches to estimate solubility and permeability in drug discovery and development settings. Adv Drug Deliv Rev. 2001;23:3–26. 10.1016/s0169-409x(00)00129-0 11259830

[60] Postigo MP, Guido RVC, Oliva G, Castilho MS, Pitta IR, de Albuquerque JFC, et al Discovery of new inhibitors of *Schistosoma mansoni* PNP by pharmacophore-based virtual screening. J Chem Inf Model. 2010;50:1693–1705. doi: 10.1021/ci100128k 20695479 10.1021/ci100128k

[61] Teague SJ, Davis AM, Leeson PD, Oprea T. The design of leadlike combinatorial libraries. Angew Chem Int Ed. 1999;38:3743–3748. 10.1002/(SICI)1521-3773(19991216)38:24<3743::AID-ANIE3743>3.0.CO;2-U 10649345

[62] Ghose AK, Viswanadhan VN, Wendoloski JJ. A knowledge-based approach in designing combinatorial or medicinal chemistry libraries for drug discovery. 1. A qualitative and quantitative characterization of known drug databases. J Comb Chem. 1999;1:55–68. 10746014 10.1021/cc9800071

[63] Oprea TI. Property distribution of drug-related chemical databases. J Comput Aid Mol Des. 2000;14:251–226. 10.1023/a:1008130001697 10756480

[64] Melo-Filho CC, Dantas RF, Braga RC, Neves BJ, Senger MR, Valente WCG, et al QSAR-driven discovery of novel chemical scaffolds active against *Schistosoma mansoni*. J Chem Inf Model. 2016;56:1357–1372. doi: 10.1021/acs.jcim.6b00055 27253773 10.1021/acs.jcim.6b00055 PMC5283162

[65] Silva Neto AM, Souza JRT, Romanello L, Cassago A, Serrao VHB, Demarco R, et al Analysis of two *Schistosoma mansoni* uridine phosphorylases isoforms suggests the emergence of a protein with a non-canonical function. Biochimie. 2016;125:12–22. doi: 10.1016/j.biochi.2016.02.007 26898674 10.1016/j.biochi.2016.02.007

[66] Angelucci F, Miele AE, Boumis G, Dimastrogiovanni D, Brunori M, Bellelli A. Glutathione reductase and thioredoxin reductase at the crossroad: the structure of *Schistosoma mansoni* thioredoxin glutathione reductase. Proteins. 2008;72:936–945. doi: 10.1002/prot.21986 18300227 10.1002/prot.21986

[67] Pereira HD, Franco GR, Cleasby A, Garratt RC. Structures for the potential drug target purine nucleoside phosphorylase from *Schistosoma mansoni* causal agent of schistosomiasis. J Mol Biol. 2005;353:584–599. doi: 10.1016/j.jmb.2005.08.045 16182308 10.1016/j.jmb.2005.08.045

[68] Torini JR, Brandão-Neto J, Demarco R, Pereira HD. Crystal structure of *Schistosoma mansoni* adenosine phosphorylase/5'-methylthioadenosine phosphorylase and its importance on adenosine salvage pathway. Plos Neglect Trop D. 2016;10:1–25. 10.1371/journal.pntd.0005178 PMC5147791 27935959

[69] Hai Y, Edwards JE, Van Zandt MC, Hoffmann KF, Christianson DW. Crystal structure of *Schistosoma mansoni* arginase, a potential drug target for the treatment of schistosomiasis. Biochemistry. 2014;53:4671–4684. doi: 10.1021/bi5004519 25007099 10.1021/bi5004519 PMC4138072

[70] Horn M, Jílková A, Vondrášek J, Marešová L, Caffrey CR, Mareš M. Mapping the pro-peptide of the *Schistosoma mansoni* cathepsin b1 drug target: modulation of inhibition by heparin and design of mimetic inhibitors. *ACS Chem Biol*. 2011;6:609–617. doi: 10.1021/cb100411v 21375333 10.1021/cb100411v

[71] Kannan S, Melesina J, Hauser A, Chakrabarti A, Heimburg T, Schmidtkunz K, et al Discovery of inhibitors of *Schistosoma Mansoni* HDAC8 by combining homology modeling, virtual screening and in vitro validation. J Chem Inf Model. 2014;54:3005–3019. doi: 10.1021/ci5004653 25243797 10.1021/ci5004653

[72] Stolfa DA, Marek M, Lancelot J, Hauser AT, Walter A, Leproult E, et al Molecular basis for the antiparasitic activity of a mercaptoacetamide derivative that inhibits histone deacetylase 8 (HDAC8) from the human pathogen *Schistosoma mansoni*. J Mol Bio. 2014;426:3442–3445. 24657767 10.1016/j.jmb.2014.03.007

