# Supplementary material for: Computational quantum chemistry, molecular docking, and ADMET predictions of imidazole alkaloids of Pilocarpus microphyllus with schistosomicidal properties
Source: PLoS One. 2018 Jun 26;13(6):e0198476. doi: 10.1371/journal.pone.0198476 (PMC6019389; doi:10.1371/journal.pone.0198476)
Supplement: S1 Table — (DOCX) [file pone.0198476.s001.docx]

**S1 Table.** Atomic charges by the Mulliken, Chelpg, and NBO methods of the epiisopiloturine, epiisopilosine, isopilosine, pilosine and macaubine alkaloids using the theoretical model B3lyp/Sdd.

|  | EPI | | | EPIIS | | | ISOP | | | PILO | | | MAC | | |
| --- | --- | --- | --- | --- | --- | --- | --- | --- | --- | --- | --- | --- | --- | --- | --- |
|  | **Chelp** | **NBO** | **Mull** | **Chelp** | **NBO** | **Mull** | **Chelp** | **NBO** | **Mull** | **Chelp** | **NBO** | **Mull** | **Chelp** | **NBO** | **Mull** |
| N1 | 0.129 | -0.430 | -0.800 | 0.138 | -0.442 | -0.171 | 0.098 | -0.442 | -0.177 | 0.119 | -0.443 | -0.168 | 0.101 | -0.441 | -0.172 |
| N2 | -0.629 | -0.556 | -0.137 | -0.597 | -0.522 | -0.088 | -0.613 | -0.525 | -0.093 | -0.635 | -0.523 | -0.090 | -0.586 | -0.522 | -0.085 |
| O1 | -0.529 | -0.576 | -0.240 | -0.537 | -0.561 | -0.240 | -0.497 | -0.570 | -0.243 | -0.527 | -0.565 | -0.235 | -0.540 | -0.569 | -0.258 |
| O2 | -0.605 | -0.593 | -0.255 | -0.595 | -0.610 | -0.269 | -0.559 | -0.584 | -0.232 | -0.601 | -0.613 | -0.272 | -0.570 | -0.581 | -0.239 |
| O3 | -0.599 | -0.809 | -0.503 | -0.685 | -0.795 | -0.469 | -0.561 | -0.819 | -0.522 | -0.692 | -0.794 | -0.473 | - | - | - |
| C1 | 0.119 | 0.230 | -0.252 | 0.176 | 0.219 | -0.250 | 0.187 | 0.215 | -0.243 | 0.211 | 0.217 | -0.258 | 0.184 | 0.221 | -0.248 |
| C2 | -0.283 | -0.403 | -0.554 | -0.435 | -0.407 | -0.567 | -0.330 | -0.414 | -0.606 | -0.448 | -0.407 | -0.571 | -0.352 | -0.408 | -0.566 |
| C3 | 0.534 | 0.133 | 0.235 | -0.133 | 0.127 | 0.387 | -0.090 | 0.131 | 0.392 | -0.160 | 0.132 | 0.370 | -0.090 | 0.126 | 0.398 |
| C4 | -0.578 | -0.456 | -0.502 | -0.140 | -0.463 | -0.527 | -0.289 | -0.466 | -0.565 | -0.092 | -0.480 | -0.476 | -0.064 | -0.490 | -0.603 |
| C5 | 0.313 | -0.229 | -0.077 | 0.184 | -0.223 | -0.067 | 0.227 | -0.224 | -0.028 | 0.157 | -0.217 | -0.138 | 0.086 | 0.024 | 0.338 |
| C6 | 0.788 | 0.835 | 0.231 | 0.855 | 0.845 | 0.236 | 0.702 | 0.838 | 0.207 | 0.851 | 0.838 | 0.244 | 0.824 | 0.794 | 0.183 |
| C7 | 0.053 | -0.343 | -0.195 | -0.065 | -0.358 | -0.232 | 0.069 | -0.342 | -0.237 | -0.015 | -0.362 | -0.227 | -0.191 | -0.121 | -0.004 |
| C8 | -0.053 | 0.146 | -0.202 | -0.109 | 0.135 | -0.244 | -0.181 | 0.144 | -0.210 | -0.124 | 0.128 | -0.229 | -0.209 | -0.659 | -0.714 |
| C9 | 0.221 | -0.081 | 0.411 | 0.257 | -0.073 | 0.468 | 0.224 | -0.082 | 0.412 | 0.309 | -0.066 | 0.475 | - | - | - |
| C10 | -0.223 | -0.217 | -0.449 | -0.233 | -0.207 | -0.443 | -0.182 | -0.229 | -0.389 | -0.247 | -0.210 | -0.458 | - | - | - |
| C11 | -0.071 | -0.210 | -0.214 | -0.066 | -0.207 | -0.213 | -0.101 | -0.207 | -0.215 | -0.071 | -0.206 | -0.211 | - | - | - |
| C12 | -0.143 | -0.212 | -0.229 | -0.126 | -0.211 | -0.227 | -0.121 | -0.209 | -0.226 | -0.130 | -0.212 | -0.228 | - | - | - |
