# Supplementary material for: Computational quantum chemistry, molecular docking, and ADMET predictions of imidazole alkaloids of Pilocarpus microphyllus with schistosomicidal properties
Source: PLoS One. 2018 Jun 26;13(6):e0198476. doi: 10.1371/journal.pone.0198476 (PMC6019389; doi:10.1371/journal.pone.0198476)
Supplement: S2 Table — (DOCX) [file pone.0198476.s002.docx]

**S2 Table.** Atomic charges by the Mulliken, Chelpg, and NBO methods of the epiisopiloturine, epiisopilosine, isopilosine, pilosine and macaubine alkaloids using the theoretical model B3lyp/6-31+G(d,p).

|  | EPI | | | EPIIS | | | ISOP | | | PILO | | | MAC | | |
| --- | --- | --- | --- | --- | --- | --- | --- | --- | --- | --- | --- | --- | --- | --- | --- |
|  | **Chelp** | **NBO** | **Mull** | **Chelp** | **NBO** | **Mull** | **Chelp** | **NBO** | **Mull** | **Chelp** | **NBO** | **Mull** | **Chelp** | **NBO** | **Mull** |
| N1 | 0.072 | -0.408 | -0.111 | 0.030 | -0.410 | -0.037 | 0.082 | -0.410 | -0.053 | 0.083 | -0.412 | -0.056 | 0.069 | -0.409 | -0.044 |
| N2 | -0.586 | -0.507 | -0.270 | -0.565 | -0.492 | -0.228 | -0.580 | -0.493 | -0.232 | -0.599 | -0.492 | -0.241 | -0.549 | -0.490 | -0.244 |
| O1 | -0.458 | -0.544 | -0.263 | -0.421 | -0.540 | -0.226 | -0.409 | -0.538 | -0.290 | -0.438 | -0.543 | -0.281 | -0.458 | -0.540 | -0.308 |
| O2 | -0.572 | -0.583 | -0.404 | -0.525 | -0.573 | -0.448 | -0.524 | -0.573 | -0.438 | -0.547 | -0.575 | -0.440 | -0.549 | -0.579 | -0.498 |
| O3 | -0.578 | -0.784 | -0.541 | -0.604 | -0.778 | -0.541 | -0.555 | -0.788 | -0.491 | -0.558 | -0.774 | -0.587 | - | - | - |
| C1 | 0.136 | 0.180 | 0.275 | 0.200 | 0.172 | 0.232 | 0.215 | 0.169 | 0.233 | 0.206 | 0.170 | 0.213 | 0.183 | 0.176 | 0.218 |
| C2 | -0.117 | -0.479 | -0.254 | -0.241 | -0.480 | -0.206 | -0.323 | -0.481 | -0.208 | -0.330 | -0.478 | -0.221 | -0.224 | -0.482 | -0.239 |
| C3 | 0.425 | 0.095 | 0.661 | -0.033 | 0.087 | 0.362 | -0.152 | 0.090 | 0.653 | -0.186 | 0.092 | 0.765 | -0.119 | 0.085 | 0.237 |
| C4 | -0.334 | -0.484 | -1.445 | -0.262 | -0.490 | -0.620 | -0.026 | -0.496 | -0.755 | 0.044 | -0.507 | -1.292 | 0.053 | -0.520 | -0.647 |
| C5 | 0.219 | -0.303 | 0.114 | 0.224 | -0.302 | 0.290 | 0.020 | -0.291 | 0.225 | 0.112 | -0.285 | 0.662 | 0.011 | 0.003 | 0.322 |
| C6 | 0.690 | 0.825 | 0.088 | 0.609 | 0.834 | 0.463 | 0.606 | 0.824 | 0.359 | 0.664 | 0.832 | 0.404 | 0.732 | 0.786 | 0.356 |
| C7 | 0.116 | -0.369 | -0.294 | 0.168 | -0.370 | -0.948 | 0.066 | -0.368 | -0.733 | 0.234 | -0.378 | -1.082 | -0.165 | -0.139 | 0.436 |
| C8 | 0.116 | 0.084 | 0.455 | 0.127 | 0.079 | 0.977 | -0.054 | 0.076 | 0.092 | -0.101 | 0.078 | 0.524 | -0.081 | -0.717 | -0.729 |
| C9 | 0.082 | -0.087 | -0.018 | 0.087 | -0.086 | -0.297 | 0.178 | -0.086 | 0.151 | 0.205 | -0.091 | 0.335 | - | - | - |
| C10 | -0.167 | -0.235 | 0.280 | -0.146 | -0.238 | 0.127 | -0.151 | -0.236 | -0.103 | -0.160 | -0.237 | 0.249 | - | - | - |
| C11 | -0.052 | -0.243 | -0.282 | -0.076 | -0.242 | -0.329 | -0.079 | -0.241 | -0.266 | -0.064 | -0.243 | -0.148 | - | - | - |
| C12 | -0.112 | -0.241 | -0.050 | -0.095 | -0.240 | -0.057 | -0.103 | -0.238 | -0.073 | -0.114 | -0.239 | -0.130 | - | - | - |
