# Supplementary material for: Computational quantum chemistry, molecular docking, and ADMET predictions of imidazole alkaloids of Pilocarpus microphyllus with schistosomicidal properties
Source: PLoS One. 2018 Jun 26;13(6):e0198476. doi: 10.1371/journal.pone.0198476 (PMC6019389; doi:10.1371/journal.pone.0198476)
Supplement: S3 Table — (DOCX) [file pone.0198476.s003.docx]

**S3 Table.** Distances of the atomic bonds and atomic angles of the epiisopiloturine, epiisopilosine, isopilosine, pilosine and macaubine alkaloids using the theoretical models B3lyp/Sdd and B3lyp/6-31+G(d,p).

| B3lyp/Sdd | EPI | EPIIS | ISOP | PILO | MAC |
| --- | --- | --- | --- | --- | --- |
| N1 – C2 | 1.465 | 1.465 | 1.468 | 1.464 | 1.466 |
| N2 – C1 | 1.337 | 1.336 | 1.336 | 1.335 | 1.336 |
| O2 = C6 | 1.233 | 1.235 | 1.231 | 1.236 | 1.233 |
| O1 – C6 | 1.390 | 1.386 | 1.392 | 1.386 | 1.412 |
| O3 – C8 | 1.469 | 1.470 | 1.477 | 1.467 |  |
| N1 – C1 – N2 | 111.662 | 111.790 | 111.805 | 111.739 | 111.804 |
| C1 – N1 – C2 | 126.834 | 125.740 | 125.649 | 125.819 | 125.989 |
| C3 – C4 – C5 | 112.642 | 113.969 | 113.886 | 113.148 | 114.253 |
| O2 = C6 - O1 | 121.997 | 121.944 | 122.046 | 122.296 | 122.714 |
| O3 – C8 – C7 | 105.378 | 110.012 | 105.924 | 111.045 |  |
| C7 – C8 – C9 | 116.002 | 115.969 | 113.873 | 114.530 |  |
| C8 – C9 – C10 | 119.049 | 118.693 | 120.005 | 119.046 |  |
| B3lyp/6-31+G(d,p) | **EPI** | **EPIIS** | **ISOP** | **PILO** | **MAC** |
| N1 – C2 | 1.454 | 1.455 | 1.455 | 1.453 | 1.455 |
| N2 – C1 | 1.317 | 1.315 | 1.316 | 1.315 | 1.316 |
| O2 = C6 | 1.207 | 1.204 | 1.206 | 1.204 | 1.209 |
| O1 – C6 | 1.356 | 1.361 | 1.358 | 1.362 | 1.373 |
| O3 – C8 | 1.432 | 1.430 | 1.440 | 1.428 | - |
| N1 – C1 – N2 | 112.267 | 112.431 | 112.411 | 112.381 | 112.450 |
| C1 – N1 – C2 | 126.972 | 125.766 | 125.847 | 125.720 | 126.070 |
| C3 – C4 – C5 | 113.358 | 114.577 | 114.417 | 113.778 | 114.432 |
| O2 = C6 - O1 | 122.220 | 121.819 | 122.093 | 121.842 | 122.692 |
| O3 – C8 – C7 | 106.392 | 106.554 | 106.229 | 107.933 | - |
| C7 – C8 – C9 | 115.138 | 115.632 | 114.224 | 114.706 | - |
| C8 – C9 – C10 | 119.205 | 118.998 | 120.419 | 119.107 | - |
