# Supplementary material for: Computational quantum chemistry, molecular docking, and ADMET predictions of imidazole alkaloids of Pilocarpus microphyllus with schistosomicidal properties
Source: PLoS One. 2018 Jun 26;13(6):e0198476. doi: 10.1371/journal.pone.0198476 (PMC6019389; doi:10.1371/journal.pone.0198476)
Supplement: S4 Table — Gibbsa, Enthalpyb, Thermalc, Energy zero pointd. (DOCX) [file pone.0198476.s004.docx]

**S4 Table.** Electrostatic energies in (Hartree) of epiisopiloturine, epiisopilosine, isopilosine, pilosine and macaubine alkaloids using the theoretical models B3lyp/Sdd, B3lyp/6-31+G(d,p) and B3lyp/6-311++G(d,p). Gibbs^a^, Enthalpy^b^, Thermal^c^, Energy zero point^d^.

|  | Energy | EPI | EPIIS | ISOP | PILO | MAC |
| --- | --- | --- | --- | --- | --- | --- |
| B3lyp/sdd | **HF^d^** | -955.243343 | -955.239355 | -955.240796 | -955.236328 | -647.912618 |
|  | **ΔT^c^** | -955.224312 | -955.220546 | -955.221980 | -955.217580 | -647.899228 |
|  | **ΔH^b^** | -955.223368 | -955.219601 | -955.221036 | -955.216636 | -647.898284 |
|  | **ΔG^a^** | -955.294028 | -955.289314 | -955.290474 | -955.286155 | -647.954685 |
| B3lyp/6-31+G(d,p) | **HF** | -955.461254 | -955.456083 | -955.458547 | -955.451371 | -648.068900 |
|  | **ΔT** | -955.442202 | -955.437099 | -955.439469 | -955.432451 | -648.055645 |
|  | **ΔH** | -955.441258 | -955.436155 | -955.438524 | -955.431507 | -648.054701 |
|  | **ΔG** | -955.511751 | -955.506116 | -955.509287 | -955.501034 | -648.110318 |
| B3lyp/6-311++G(d,p) | **HF** | -955.663191 | -955.657935 | -955.660326 | -955.653363 | -648.207347 |
|  | **ΔT** | -955.644630 | -955.638914 | -955.641184 | -955.634424 | -648.194075 |
|  | **ΔH** | -955.643686 | -955.637970 | -955.640239 | -955.633480 | -648.193131 |
|  | **ΔG** | -955.711130 | -955.708003 | -955.711291 | -955.703143 | -648.248798 |
