# Supplementary material for: Computational quantum chemistry, molecular docking, and ADMET predictions of imidazole alkaloids of Pilocarpus microphyllus with schistosomicidal properties
Source: PLoS One. 2018 Jun 26;13(6):e0198476. doi: 10.1371/journal.pone.0198476 (PMC6019389; doi:10.1371/journal.pone.0198476)
Supplement: S5 Table — (DOCX) [file pone.0198476.s005.docx]

**S5 Table.** Molecular orbital values HOMO and LUMO (in eV) of epiisopiloturine, epiisopilosine, isopilosine, pilosine and macaubine alkaloids using the theoretical models B3lyp/Sdd, B3lyp/6-31+G(d,p) and B3lyp/6-311++G(d,p).

|  | |  | EPI | EPIIS | ISOP | PILO | MAC |
| --- | --- | --- | --- | --- | --- | --- | --- |
| B3lyp/Sdd | **HOMO** | -4 | -7.249 | -7.634 | -7.368 | -7.542 | -7.755 |
|  |  | -3 | -7.129 | -7.190 | -7.219 | -7.207 | -7.722 |
|  |  | -2 | -6.932 | -7.187 | -7.096 | -7.091 | -7.662 |
|  |  | -1 | -6.709 | -7.096 | -6.946 | -7.086 | -7.280 |
|  |  | 0 | -6.486 | -6.558 | -6.269 | -6.439 | -6.702 |
|  | **GAP** |  | 5.984 | 5.700 | 5.399 | 5.565 | 5.028 |
|  | **LUMO** | 0 | -0.502 | -0.858 | -0.870 | -0.873 | -1.673 |
|  |  | +1 | -0.281 | -0.709 | -0.623 | -0.698 | -0.022 |
|  |  | +2 | 0.029 | -0.549 | -0.478 | -0.562 | 1.019 |
|  |  | +3 | 0.103 | 0.083 | 0.297 | 0.167 | 1.391 |
|  |  | +4 | 1.158 | 1.120 | 1.362 | 1.233 | 1.721 |
| B3lyp/6-31+G(d,p) | **HOMO** | -4 | -7.625 | -7.637 | -7.530 | -7.565 | -7.839 |
|  |  | -3 | -7.271 | -7.525 | -7.440 | -7.464 | -7.791 |
|  |  | -2 | -6.959 | -7.174 | -7.214 | -7.255 | -7.695 |
|  |  | -1 | -6.752 | -6.960 | -7.080 | -7.067 | -7.645 |
|  |  | 0 | -6.450 | -6.409 | -6.282 | -6.313 | -6.613 |
|  | **GAP** |  | 5.877 | 5.621 | 5.390 | 5.471 | 5.103 |
|  | **LUMO** | 0 | -0.573 | -0.788 | -0.891 | -0.841 | -1.510 |
|  |  | +1 | -0.382 | -0.603 | -0.645 | -0.717 | -0.562 |
|  |  | +2 | -0.288 | -0.589 | -0.423 | -0.673 | -0.139 |
|  |  | +3 | -0.181 | -0.235 | -0.273 | -0.153 | 0.024 |
|  |  | +4 | -0.160 | -0.030 | -0.068 | -0.085 | 0.090 |
| B3lyp/6-311++G(d,p) | **HOMO** | -4 | -7.670 | -7.673 | -7.568 | -7.607 | -7.881 |
|  |  | -3 | -7.311 | -7.548 | -7.457 | -7.485 | -7.825 |
|  |  | -2 | -7.006 | -7.230 | -7.271 | -7.302 | -7.750 |
|  |  | -1 | -6.798 | -7.011 | -7.134 | -7.100 | -7.673 |
|  |  | 0 | -6.493 | -6.453 | -6.326 | -6.359 | -6.654 |
|  | **GAP** |  | 5.851 | 5.619 | 5.382 | 5.462 | 5.133 |
|  | **LUMO** | 0 | -0.641 | -0.833 | -0.943 | -0.897 | -1.520 |
|  |  | +1 | -0.555 | -0.746 | -0.700 | -0.832 | -0.739 |
|  |  | +2 | -0.457 | -0.651 | -0.614 | -0.730 | -0.247 |
|  |  | +3 | -0.313 | -0.372 | -0.314 | -0.296 | -0.160 |
|  |  | +4 | -0.256 | -0.213 | -0.285 | -0.272 | -0.118 |
