# Supplementary material for: Computational quantum chemistry, molecular docking, and ADMET predictions of imidazole alkaloids of Pilocarpus microphyllus with schistosomicidal properties
Source: PLoS One. 2018 Jun 26;13(6):e0198476. doi: 10.1371/journal.pone.0198476 (PMC6019389; doi:10.1371/journal.pone.0198476)
Supplement: S6 Table — Atom labels accordingly to S5 Fig. (DOCX) [file pone.0198476.s006.docx]

**S6 Table.** epiisopiloturine, epiisopilosine, isopilosine, pilosine and macaubine ^13^C NMR chemical shifts. Atom labels accordingly to S5 Fig.

| Atom | EPI | EPIIS | ISOP | PILO | MAC |
| --- | --- | --- | --- | --- | --- |
| C6 | 181.55 | 177.41 | 179.13 | 176.69 | 177.56 |
| C3 | 151.08 | 132.76 | 134.34 | 134.76 | 132.15 |
| C9 | 149.65 | 148.26 | 149.71 | 150.43 | - |
| C1 | 143.13 | 142.83 | 142.51 | 142.36 | 143.21 |
| C13 | 134.45 | 133.63 | 133.02 | 134.56 | - |
| C14 | 134.30 | 134.99 | 136.43 | 135.56 | - |
| C12 | 133.89 | 133.10 | 133.43 | 133.22 | - |
| C11 | 133.20 | 132.32 | 133.26 | 132.20 | - |
| C10 | 131.14 | 129.78 | 130.08 | 130.30 | - |
| C16 | 122.08 | 135.82 | 134.94 | 136.72 | 135.64 |
| C8 | 85.56 | 77.69 | 83.10 | 79.14 | 9.85 |
| C15 | 73.29 | 72.73 | 73.78 | 69.57 | 74.13 |
| C7 | 57.65 | 54.70 | 55.32 | 53.35 | 131.01 |
| C5 | 47.76 | 42.15 | 46.13 | 45.54 | 162.38 |
| C4 | 37.39 | 29.67 | 32.16 | 26.81 | 26.07 |
| C2 | 33.04 | 31.51 | 31.30 | 31.41 | 31.47 |
