# Supplementary material for: Computational quantum chemistry, molecular docking, and ADMET predictions of imidazole alkaloids of Pilocarpus microphyllus with schistosomicidal properties
Source: PLoS One. 2018 Jun 26;13(6):e0198476. doi: 10.1371/journal.pone.0198476 (PMC6019389; doi:10.1371/journal.pone.0198476)
Supplement: S7 Table — (DOCX) [file pone.0198476.s007.docx]

**S7 Table.** Dipole moment in epiisopiloturine, epiisopilosine, isopilosine, pilosine and macaubine alkaloids using the theoretical models B3lyp/Sdd, B3lyp/6-31+G(d,p), and B3lyp/6-311++G(d,p).

|  | B3lyp/Sdd | B3lyp/6-31+G(d,p) | B3lyp/6-311++G(d,p) |
| --- | --- | --- | --- |
| EPI | 6.296453 | 5.863899 | 5.866797 |
| EPIIS | 4.706137 | 5.761462 | 5.717937 |
| ISOP | 6.158533 | 5.347280 | 5.317699 |
| PILO | 7.217896 | 7.437599 | 7.371430 |
| MAC | 4.953716 | 4.558397 | 4.523608 |
