# Supplementary material for: Computational quantum chemistry, molecular docking, and ADMET predictions of imidazole alkaloids of Pilocarpus microphyllus with schistosomicidal properties
Source: PLoS One. 2018 Jun 26;13(6):e0198476. doi: 10.1371/journal.pone.0198476 (PMC6019389; doi:10.1371/journal.pone.0198476)
Supplement: S8 Table — (DOCX) [file pone.0198476.s008.docx]

**S8 Table.** Infrared spectroscopic frequencies (cm^-1^) of the epiisopiloturine, epiisopilosine, isopilosine, pilosine and macaubine alkaloids and their assignments using the theoretical model B3lyp/6-311++G(d,p).

| **EPI** | **EPIIS** | **ISOP** | **PILO** | **MAC** | **Assignment** |
| --- | --- | --- | --- | --- | --- |
| 390 | 281 | 317 | 261 | - | rotation in H-O |
| 1162 | 1159 | 1037 | 1154 | 1075 | stretching in plane in dihydrofuran |
| 1303 | 1308 | 1312 | 1306 | - | stretching C-C in benzene |
| 1318 | 1364 | 1368 | 1371 | 1369 | stretching C-N in imidazole |
| 1408 | 1416 | 1414 | 1417 | 1413 | symmetric stretching N1-C1-N2 in imidazole |
| 1455 | 1456 | 1455 | 1455 | 1454 | stretching in CH_3_ (C2-N1) in imidazole |
| 1480 | 1484 | 1485 | 1485 | - | stretching C-C in benzene |
| 1488 | 1492 | 1484 | 1490 | 1489 | rotation in CH_3_ (C2) in imizadole |
| 1525 | 1524 | 1523 | 1523 | 1523 | symmetric stretching in N-C-N in Imidazole |
| 1578 | 1586 | 1585 | 1585 | 1584 | stretching C-C in imidazole |
| 1612 | 1624 | 1625 | 1623 | - | stretching C-C in benzene |
| 1631 | 1643 | 1643 | 1641 | - | stretching C-C in benzene |
| 1785 | 1846 | 1838 | 1845 | 1825 | symmetric stretching C=O |
| 2963 | 2961 | 2998 | 2954 | - | stretching in H-C8 |
| 3022 | 3039 | 3035 | 3035 | 3039 | stretching in H-C2 |
| 3783 | 3830 | 3820 | 3830 | - | stretching in H-O3 |
